# An Integrated Approach to Elucidate the Intra-Viral and Viral-Cellular Protein Interaction Networks of a Gamma-Herpesvirus

**DOI:** 10.1371/journal.ppat.1002297

**Published:** 2011-10-20

**Authors:** Shaoying Lee, Lukasz Salwinski, Chaoying Zhang, Derrick Chu, Claire Sampankanpanich, Nichole A. Reyes, Abbey Vangeloff, Fangfang Xing, Xudong Li, Ting-Ting Wu, Sudhir Sahasrabudhe, Hongyu Deng, Douglas J. LaCount, Ren Sun

**Affiliations:** 1 School of Dentistry, University of California Los Angeles, Los Angeles, California, United States of America; 2 Department of Molecular and Medical Pharmacology, University of California Los Angeles, Los Angeles, California, United States of America; 3 UCLA DOE-Institute for Genomics and Proteomics, University of California Los Angeles, Los Angeles, California, United States of America; 4 Department of Medicinal Chemistry and Molecular Pharmacology, Purdue University, West LaFayette, Indiana, United States of America; 5 Department of Molecular Cell and Developmental Biology, University of California Los Angeles, Los Angeles, California, United States of America; 6 Prolexys Pharmaceuticals, Salt Lake City, Utah, United States of America; 7 Institute of Biophysics, Chinese Academy of Sciences, Beijing, China; University of Florida, United States of America

## Abstract

Genome-wide yeast two-hybrid (Y2H) screens were conducted to elucidate the molecular functions of open reading frames (ORFs) encoded by murine γ-herpesvirus 68 (MHV-68). A library of 84 MHV-68 genes and gene fragments was generated in a Gateway entry plasmid and transferred to Y2H vectors. All possible pair-wise interactions between viral proteins were tested in the Y2H assay, resulting in the identification of 23 intra-viral protein-protein interactions (PPIs). Seventy percent of the interactions between viral proteins were confirmed by co-immunoprecipitation experiments. To systematically investigate virus-cellular protein interactions, the MHV-68 Y2H constructs were screened against a cellular cDNA library, yielding 243 viral-cellular PPIs involving 197 distinct cellar proteins. Network analyses indicated that cellular proteins targeted by MHV-68 had more partners in the cellular PPI network and were located closer to each other than expected by chance. Taking advantage of this observation, we scored the cellular proteins based on their network distances from other MHV-68-interacting proteins and segregated them into high (Y2H-HP) and low priority/not-scored (Y2H-LP/NS) groups. Significantly more genes from Y2H-HP altered MHV-68 replication when their expression was inhibited with siRNAs (53% of genes from Y2H-HP, 21% of genes from Y2H-LP/NS, and 16% of genes randomly chosen from the human PPI network; p<0.05). Enriched Gene Ontology (GO) terms in the Y2H-HP group included regulation of apoptosis, protein kinase cascade, post-translational protein modification, transcription from RNA polymerase II promoter, and IκB kinase/NFκB cascade. Functional validation assays indicated that PCBP1, which interacted with MHV-68 ORF34, may be involved in regulating late virus gene expression in a manner consistent with the effects of its viral interacting partner. Our study integrated Y2H screening with multiple functional validation approaches to create γ-herpes viral-viral and viral-cellular protein interaction networks.

## Author Summary

Persistent infections by the herpesviruses Epstein Barr virus (EBV) and Kaposi's sarcoma herpesvirus (KSHV) are associated with tumor formation. To better understand how these and other related viruses interact with their host cells to promote virus replication and cause disease, we studied murine gamma-herpesvirus 68 (MHV-68). MHV-68 belongs to the same group of herpesviruses as EBV and KSHV, but has the advantage of being able to replicate efficiently in cell culture. Our study used genome-wide screens to identify 23 protein-protein interactions between the 80 MHV-68 proteins. Several of these interactions are likely to be important for assembling new viruses. We also discovered 243 interactions between MHV-68 and cellular proteins. To help prioritize cellular proteins for follow up studies, we developed a new computational tool to analyze our data. Proteins with high priority scores were more likely to affect viral replication than low priority proteins. Among the cellular proteins that had the greatest effect on MHV-68 replication was PCBP1, which negatively regulated MHV-68 late gene expression. This study identified many novel cellular proteins involved in MHV-68 replication and established a method to identify important proteins from high-throughput virus-cellular protein-protein interaction data sets.

## Introduction

Gamma-herpesviruses comprise a subfamily of *Herpesviridae*, a group of enveloped, double-stranded DNA viruses with large and complex genomes ranging from 120 to 230 kbp in length [1]. Herpesviruses have two distinct life cycle phases: latency and lytic replication. During latent infection, no active viral replication occurs and only a limited number of viral genes, including non-coding RNAs, membrane proteins, and various nuclear antigens, are expressed to maintain the viral genome and guard against host immune responses [2]–[4]. Upon reactivation of the latent virus, lytic replication ensues and results in the production of viral progeny, leading to the destruction of the host cell. In contrast to alpha and beta herpesviruses, gamma-herpesviruses have distinct cellular tropisms and establish life-long persistent infections in lymphocytes [5]. Two well-known human gamma-herpesviruses, Kaposi's sarcoma herpesvirus (KSHV) and Epstein Barr Virus (EBV), are associated with the development of both lymphoid and non-lymphoid cell tumors, including Kaposi's sarcoma (KS), Burkitt's lymphoma, nasopharyngeal carcinoma, and lymphoproliferative diseases in immunocompromised patients [5]–[8]. Though tumorigenesis induced by gamma-herpesviruses requires multiple genes expressed during latent infection, it has been suggested that lytic viral gene products expressed during sporadic reactivation in tumor lesions also promote cell growth [4], [9], [10]. A better understanding of the interplay between gamma-herpesviruses and the cells they replicate in will provide insight into the tumor-promoting properties of herpesviruses and may lead to the development of improved therapeutic strategies.

MHV-68, a natural rodent pathogen, serves as a useful model for the study of human gamma-herpesviruses due to the similarity of the gene sequences and genome organization. 80% of MHV-68 ORFs share significant homology with their human viral counterparts. Furthermore, the ability of MHV-68 to establish robust *de novo* productive infections in various human and mouse cell lines and to infect laboratory mice provides an experimental system to study the biological significance of virus-host cell interactions *in vitro* and *in vivo* [11]–[13]. The recent development of an MHV-68 virus that expresses luciferase provides a powerful tool to monitor virus replication in live mice [14]. Although it was known that MHV-68 infected multiple tissue types, live imaging of viral replication revealed new sites of infection and demonstrated that timing of lytic replication and clearance varied among different tissues and organs [14]–[16]. Combined with the ability to silence gene expression by RNA interference, and the availability of transgenic and knockout mice, a wide range of tools are available to interrogate MHV-68-host interactions both in vitro and in vivo.

Over the past several years, we have undertaken multiple studies to systematically characterize the genes and proteins of MHV-68, beginning with a comprehensive analysis of viral gene expression [17] and extending to proteomic analysis of the MHV-68 virion [18], large-scale signature tagged mutagenesis to identify essential MHV-68 open reading frames [19], and high-throughput random insertional mutagenesis for genome-scale functional profiling [20]. In this report we characterize the intra-viral and virus-cellular protein interaction networks of MHV-68. Protein-protein interactions are critical for the functions of most proteins, and the systematic identification of viral protein interactions will provide insight into MHV-68 replication and pathogenesis. For example, viral proteins must interact with each other to create complexes needed for genome replication and virus assembly. However, even viruses with large genomes, such as the herpesviruses, require cellular proteins to supply activities not encoded in the viral genome. Viruses also interact with cellular proteins to manipulate cellular pathways in order to promote an environment favorable to the virus. Conversely, the cell expresses proteins that may bind directly to viral proteins to inhibit their functions to promote an antiviral state.

**Figure 1:**
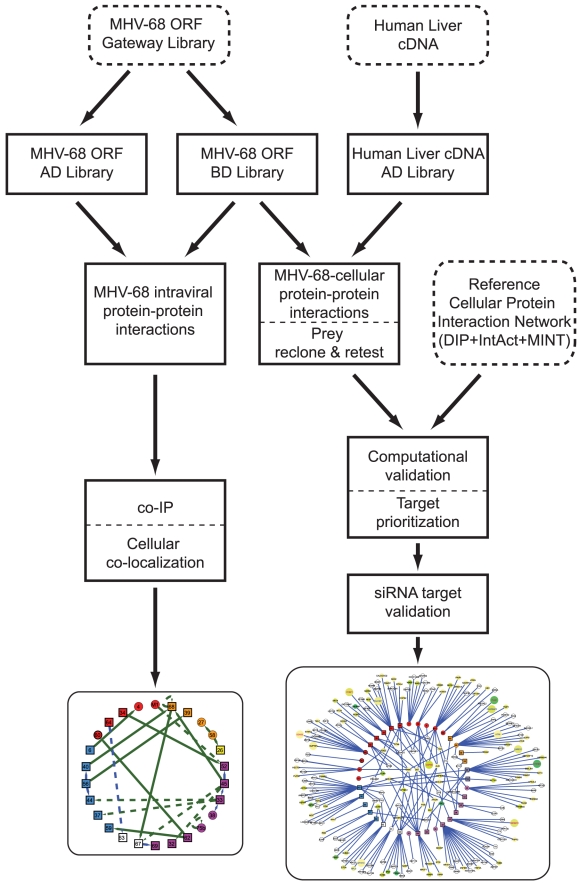
Overview of the generation and validation of the MHV-68 intra-viral and virus-cellular protein-protein interaction networks.

Although several recent studies have extensively characterized the intra-viral protein interactions of five herpesviruses [21]–[25] much less is known about the gamma-herpesviral-cellular PPI network. To date, a single experimental study reported 173 interactions between EBV and human proteins [24] and one computational analysis predicted 20 herpesvirus-cellular protein interactions [21]. These studies are further limited by the lack of robust replication of human herpesvirus *in vitro* and *in vivo*, which makes functional validation of virus-cellular interactome difficult. MHV-68 complements this deficiency and has the capacity for effective genetic manipulation to study lytic replication *in vitro* [19], [26]. Thus, a comprehensive inventory of MHV-68 protein interactions will provide a valuable resource to understand the interplay between the virus and host cell and will yield insights into the functions of individual proteins during viral replication. Therefore, to elucidate the molecular interactions of MHV-68 proteins we conducted genome-wide Y2H screens (Fig. 1). We report the identification of 23 intra-viral protein interactions and 243 virus-cellular protein interactions, the vast majority of which are novel. Evaluation of the virus-cellular protein interactions was aided by the development of a novel scoring method based on the network distances of MHV-68-interacting cellular proteins within a high-confidence binary cellular protein interaction network. Our study integrated computational analysis of MHV-68 interacting proteins with multiple complementary functional assays to demonstrate the biological relevance of the MHV-68 intra-viral and virus-cellular protein interactomes.

## Results

### Genome-wide Y2H screens for MHV-68 intra-viral protein-protein interactions

**Figure 2:**
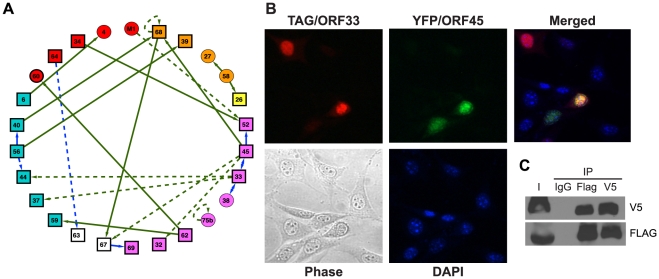
The network of interactions between MHV-68 proteins. (A) The MHV-68 intra-viral protein interaction network. Rectangles designate viral proteins essential for MHV-68 lytic replication; circles, nonessential MHV-68 proteins. Colors indicate protein functions (blue, DNA replication complex; red, regulatory proteins; orange, envelope proteins; purple, capsid; magenta, assembly or egress; white, unknown function). Arrows point from baits to preys. Interactions confirmed by co-immunoprecipitation (co-IP) are shown as solid lines, whereas those found only by Y2H are shown as dashed lines. Green lines indicate novel interactions found in this study; blue lines represent interactions detected between homologues in other data sets (see Table S1 for references). (B) ORF33 and ORF45 co-localize in the nucleus. Expression plasmids encoding FLAG epitope-tagged ORF33 and YFP-tagged ORF45 were co-transfected into NIH 3T3 cells. FLAG-ORF33 was detected by IFA with anti-FLAG-M2 primary antibody and Alexa Fluor 594-conjugated anti-mouse IgG antibodies. Panel 1 shows FLAG epitope-tagged ORF33 (red); panel 2, YFP-ORF45 (green); panel 3, merged image; panel 4, phase contrast image of same field; panel 5: DAPI-stained nuclei. (C) Co-IP of ORF33 and ORF45. FLAG epitope-tagged ORF33 and V5 epitope-tagged ORF45 were co-expressed in 293T cells, immunoprecipitated with either control IgG, anti-FLAG M2 or anti-V5 antibodies, and subjected to western blotting with anti-V5 antibody (upper panel) and anti-FLAG (bottom panel). I indicate input.

To systematically investigate the intra-viral PPI networks, genome-wide Y2H screens with MHV-68 genes were performed (Fig. 1). All predicted viral ORFs [12] were cloned into a Gateway entry vector, enabling efficient shuttling of genes into different expression plasmids. ORFs larger than 1.5 kb were divided into smaller fragments clones since large genes tend to yield fewer interactions in the Y2H assay [27]. Similarly, genes encoding proteins with predicted trans-membrane domains were truncated to express only the soluble portion. The entire library was then transferred into the Y2H DNA-binding domain (pDEST32) and activation domain (pDEST22) destination vectors and transformed into the yeast strains PJ69-4α and PJ69-4a, respectively [28]. All possible pair-wise combinations were tested independently for activation of the reporter genes *HIS3* and *ADE2* in quadruplicate using a 384-spot array format. Twenty-five (25) pairs of MHV-68 proteins were found to activate both reporter genes (Fig. 2A, Table S1), including two pairs in which the partners interacted when cloned as either bait or prey.

The physical interactions between viral proteins were validated by co-immunoprecipitation (Co-IP) and co-localization assays (Table S1; Fig. 2B and C). MHV-68 genes and gene fragments were transferred to mammalian expression vectors as fusions to the epitope tags FLAG (pTAG) and V5 (pHB) or the fluorescent proteins GFP and RFP. HEK293T cells were transfected with pairs of plasmids encoding putative interacting proteins and infected with MHV-68 24 h later. Cell lysates were co-immunoprecipitated with anti-FLAG, anti-V5, or non-specific anti-mouse IgG antibodies and subjected to western blotting with anti-FLAG and anti-V5 antibodies. Of the 23 intra-viral interactions, 16 (70%) pairs were confirmed in at least one direction of the antibody pull-down (Table S1, Fig. S1). These interactions were further validated by co-localization of the interacting partners using either pairs of GFP- and RFP-tagged or FLAG- and V5-epitope tagged proteins expressed in NIH 3T3 cells (Table S1). Three additional interactions could not be confirmed due to the low expression in our system, but were previously reported between homologous proteins from other herpesvirus family members (Table S1) [29]–[33]. In total, 19 out of 23 interactions were supported by secondary experiments for a combined confirmation rate of 83%.

To illuminate the biological roles of the newly identified intra-viral PPIs, interactions were grouped according to the known and predicted functions of the viral proteins (viral DNA replication, assembly/egress, capsid and envelope structural proteins, and unknown) (Fig. 2A). Tegument proteins were prominently featured among the interactions detected under our stringent screening conditions. In particular, ORF45 and ORF33 interacted strongly in both orientations in the Y2H assay, co-localized in the nucleus (Fig. 2B), and co-purified in both directions in antibody pull-down experiments (Fig. 2C). Both are tegument proteins expressed late during the infection cycle [34], [35] and are essential for viral assembly and egress. Their homologues in KSHV interact with KSHV ORF64, a hub protein that recruits other tegument proteins [22]. Our results suggest that the three proteins - ORF33, ORF45 and ORF64 - may be a part of a larger multi-protein complex (Fig. S1-B).

To develop a comprehensive view of the current status of gamma-herpesvirus intra-viral protein interactions, we integrated the large-scale and literature-curated EBV and KSHV intra-viral protein interactions from [25] with the MHV-68 interactions identified here (Fig. S2). Seven non-self MHV-68 interactions were shared with each gamma-herpes virus, but only two interactions were found in all three (Table S1). Though many more interactions have been identified in both EBV and KSHV, the extent of the overlap between the two is surprisingly small, with only 13 interactions in common (5% of the 250 non-self interactions in EBV; 7% of the 175 non-self interactions in KSHV). The relatively low overlap between EBV and KSHV appears to be due to different proteins from KSHV and EBV yielding interactions in the various screens (Fig. S2). Assuming that interactions between conserved gamma-herpesviruses are conserved, this suggests that the screens for gamma-herpesvirus intra-viral interactions have missed many interactions.

### Genome-wide Y2H screens for MHV-68-cellular protein-protein interactions

To identify cellular proteins that interacted with MHV-68 proteins, we performed Y2H library screens with 84 MHV-68 constructs. Since our primary goal in this study was to identify host factors that played a role in MHV-68 lytic replication, we chose a high quality cDNA library from a tissue type (human liver) known to support MHV-68 infection ([36]and unpublished data). MHV-68 undergoes lytic replication in various human and mouse cell lines in *vitro*, including liver-derived cell lines ([11]–[13], and unpublished data). In addition, MHV-68 productively infects the liver in experimentally inoculated mice [14]–[16]. Finally, liver tissue contains multiple cell types and expresses a broad range of cellular genes. We acknowledge that by using a human cDNA library, interactions with murine-specific proteins will be missed.

**Figure 3:**
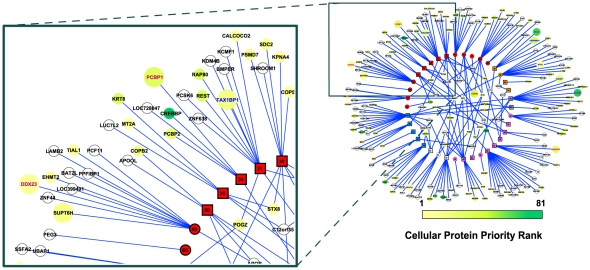
The MHV-68-cellular protein interactome. Viral proteins (rectangles) are displayed as a ring and are grouped according to their functional annotation (colors are as described in legend to Fig. 2). Cellular proteins (circles) that interacted with one viral protein are located outside of the ring, whereas those that interacted with multiple viral proteins are located inside. Cellular proteins are colored according to the priority rank, with unranked proteins are shown in white. The size of the circle indicates the magnitude of the effect on MHV-68 lytic replication caused by inhibiting the expression of the cellular gene by RNAi. Large circles imply greater effect; small circle, no effect or not tested. Font color of the cellular protein label indicates the direction of the effect on MHV-68 lytic replication (red, inhibiting expression of the cellular gene increased replication, dark green; inhibiting expression of the cellular gene decreased replication, black, no effect or not tested).

Our Y2H screens employed a modified Y2H vector in which gene fragments are cloned between the *GAL4* activation domain and *URA3*. (Fig. 1) [37], [36]. Growth of yeast on medium lacking uracil selects for inserts that are cloned in frame and expressed, and selects against clones whose inserts are in the wrong reading frame, contain stop codons, or are poorly expressed. Thus, the library has fewer clones than traditional libraries, which increases the likelihood of comprehensively sampling the AD clones in Y2H screens. In addition, frame-shift mutations that occur during Y2H screening – an important source of false-positives [38] – are selected against. The 84 MHV-68 genes and gene fragments described above were cloned into the *Gal4* DNA binding domain plasmid pXDGATcy86 and screened at least twice against the human liver Y2H library (215 screens total). Seventy-four MHV-68 genes yielded positive colonies in at least one screen. The activation domain inserts from 1879 colonies (up to 48 per screen) were PCR-amplified and sequenced, yielding 1544 pairs of interacting proteins representing 508 different interactions. All unique cellular gene fragments (excluding known false-positives) were then re-cloned into the activation domain plasmid in fresh yeast cells and retested in the Y2H assay with the bait from the original Y2H assay. Two hundred-forty three pairs independently activated expression of both the *HIS3* and *ADE2* reporter genes in the retest and were considered true Y2H interactions (Fig. 3A, Fig. S3, Table S2). Another 25 pairs activated expression of only one Y2H reporter.

### MHV-68 proteins bind to cellular proteins with similar functions

To characterize the cellular factors identified in the Y2H screen, we grouped them according to the known or predicted functions of their viral partners (DNA replication, life-cycle regulation, virion assembly/egress, and capsid or envelope structural proteins) and analyzed the functional annotation of all cellular proteins and their immediate neighbors in the cellular PPI network. Interestingly, the cellular factors identified in the screen were enriched in Gene Ontology (GO) terms related to the function of their viral partners (Fig. S4A). For example, the targets of the viral DNA replication proteins participated in DNA replication, recombination, and repair. Similarly, viral regulatory proteins tended to interact with cellular proteins involved in the regulation of ubiquitin-ligase/protein kinase activities, protein amino acid phosphorylation, and the antigen receptor and integrin-mediated signaling pathways, whereas viral assembly/egress proteins tended to interact with cellular proteins involved in cell adhesion, lipid homeostasis, and regulation of cytoskeleton organization/biogenesis (Fig. S4A). GO terms related to regulation of apoptosis and I-kB kinase/NF-kB cascade were enriched among the partners of viral proteins in multiple functional categories (DNA replication, regulation, and structural) while the regulation of post-translational modification term was enriched in all the functional groups. This latter observation may reflect the importance of these modifications at multiple stages of the viral replication cycle.

### Network context of the cellular factors that interacted with MHV-68 proteins

**Figure 4:**
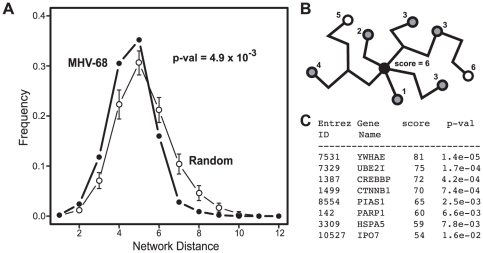
Network context of cellular proteins that interacted with MHV-68 proteins. (A) Distribution of the network distances between cellular proteins that interacted with MHV-68 proteins. The distances between cellular proteins that interacted with MHV-68 proteins in the reference cellular protein interaction network (i.e., the number of protein-protein interactions that must be passed through to connect a pair of proteins) were calculated and plotted. Closed circles (thick line) represent the observed distribution of distances between the MHV-68-interacting proteins. Bars (thin line) show the average distance distribution from an equivalent number of randomly selected cellular proteins. (B) Calculation of a network neighborhood-based priority score. The black circle indicates a cellular protein that interacted with an MHV-68 protein and that served as the starting point in this example. Gray circles indicate other human proteins that interacted with MHV-68 proteins. White circles represent human proteins that did not interact with MHV-68. The priority score was calculated by counting the cellular proteins that interacted with MHV-68 proteins within a distance of four interactions. (C) The priority scores of the top ranked cellular proteins identified in the Y2H screen.

In order to analyze the network properties of the viral-cellular protein interactions, cellular proteins identified in the Y2H screens were mapped onto the nodes of a high-confidence cellular PPI network. This network consisted of binary interactions reported in the DIP [39], IntAct [40] and MINT [41] databases that were supported by at least one small-scale or multiple high-throughput experiments (see Methods for details). Of the 197 distinct cellular proteins identified in our screen, 101 were present in this reference network. The cellular proteins identified in the screen were connected to an average of 5.7 neighbors, a number significantly larger than expected by chance (4.1; p-value 1.8×10^−2^). In addition, the average network distance between two proteins that interacted with MHV-68 proteins, calculated as the length of the shortest path connecting the corresponding network vertices, was smaller than expected for two proteins picked randomly from the reference network (4.6 versus 5.3; p-value 4.0×10^−4^). Similar trends were also observed with cellular proteins that interacted with EBV [24] (Table 1). Graphing the distribution of network distances between proteins targeted by MHV-68 revealed a significant shift toward smaller values as compared to the distribution obtained for an equally-sized set of proteins randomly selected from the reference network (p-value 4.9×10^−3^) (Fig. 4A). The distribution of the experimental data displayed a sharp drop-off at the large inter-protein distances and a higher frequency of proteins three or fewer edges away from each other. These observations indicate that the cellular factors that interacted with MHV-68 proteins are located nearer to each other than expected by chance within the reference PPI network.

**Table 1:** Network properties of cellular proteins that interacted with MHV-68 proteins.

Dataset	MHV-68	EBV(Calderwood 2007)	Reference Network
Distinct Target Proteins	197 (101*)	113 (65*)	4280
Protein Degree (p-value)	5.70+/−7.36(1.84×10^−2^)	4.487+/−5.60(2.88×10^−1^)	4.11+/−6.48
Network Distance(p-value)	4.63+/−1.13(4.0×10^−4^)	4.74+/−1.24(1.04×10^−2^)	5.30+/−1.47

*Number of proteins found in the reference cellular protein interaction network.

In order to rule out the possibility that the observed distribution arose as a result of biases in the dataset, we performed two additional analyses. One potential source of bias is the composition of the Y2H AD library. Since the library was not normalized, highly expressed genes are likely overrepresented and may give rise to more interactions, including potential false-positive interactions. We therefore analyzed how sensitive the distribution of network distances was to the removal of cellular proteins that interacted with more than one viral protein. As we observed no significant changes (Fig. S5A) we infer that, even if present, compositional bias of the cDNA library does not affect the inter-target distance distribution. A second potential source of bias is the presence of highly connected proteins (i.e., those with high degree), which are enriched among the partners of MHV-68 proteins. Such proteins, by virtue of the large number of cellular proteins they interact with, have a greater likelihood of being close to another MHV-68-binding protein by chance. To test this possibility, we compared the average distribution of network distances between a set of randomly selected proteins with that obtained for an equivalently sized set of proteins having the same degree distribution of the cellular proteins that interacted with MHV-68. Although the distribution of distances in the degree-modified set of random proteins was shifted slightly toward shorter path lengths (Fig. S5B), the shift was less than that observed in the experimental data. Also, the difference between the experimentally observed distance distribution and the one obtained for the modified reference set remained statistically significant (p-value 4.8×10^−2^). We therefore concluded that the distinct shape of the distance distribution histogram was an inherent property of the set of cellular proteins specifically targeted by the MHV-68 virus, and was not influenced by the potential composition bias of the cDNA library used in our screen or higher than average connectivity of the targeted cellular proteins. Similar changes in the distance distribution profiles were observed in previously reported sets of proteins identified in viral-cellular protein interaction screens (Fig. S6), suggesting that this may be a general feature of cellular proteins targeted by viruses.

### Functional validation of the cellular partners of MHV-68 proteins

Since the shift toward shorter distances between cellular proteins that interacted with MHV-68 was unlikely to have occurred by chance, we hypothesized that interactions with closely linked cellular proteins was important for MHV-68 replication. We further reasoned that cellular proteins that bound to MHV-68 proteins and that were located in regions of the cellular protein interaction network with higher densities of MHV-68 targeted proteins were more likely to play an important role in the MHV-68 life cycle. To test this hypothesis, we developed a scoring system to identify such proteins. For each cellular protein that interacted with an MHV-68 protein, we counted the number of other cellular proteins that bound to an MHV-68 protein and that were within four protein-protein interactions in the cellular protein interaction network (see example in Fig. 4B). The choice of the limiting distance was based on computational analyses indicating that functional correlations within PPI networks tend to disappear at network distances above four [42], [43]. Each of the 101 cellular proteins that interacted with an MHV-68 protein and that were present in the reference cellular protein interaction network were scored as described above, with scores ranging from 0 to 81. To estimate the statistical significance of this score, p-values corresponding to the probability of having that many neighboring viral targets were calculated (Fig. 4C, Table S3). We then divided the proteins into three groups based on their network distance scores: high-priority (Y2H-HP, which included 60 top ranked genes), low priority (Y2H-LP, which included the 51 proteins with the lowest scores), and not scored (Y2H-NS, which included the 96 proteins not present in the reference cellular protein interaction network).

As a preliminary test to evaluate the biological relevance of prioritization scores, we inhibited the expression of the 60 highest scoring cellular genes by RNA interference and analyzed the effect on MHV-68 replication. A two-step luciferase reporter virus system was employed to monitor viral replication after siRNA treatment in a sensitive and medium throughput manner [14], [44]. First, siRNA-treated 293T cells were infected with M3-Luc MHV-68, a reporter virus that contains an M3-promoter driven firefly luciferase reporter. Second, supernatant from the infected siRNA-treated cells was used to infect fresh 293T cells and luciferase levels were measured 20 h later. Because luciferase expression from the M3-Luc virus has a linear relationship across a broad range of the infectious particles (Fig. S7A), the level of luciferase activity provides an indirect measurement of viral titer.

**Figure 5:**
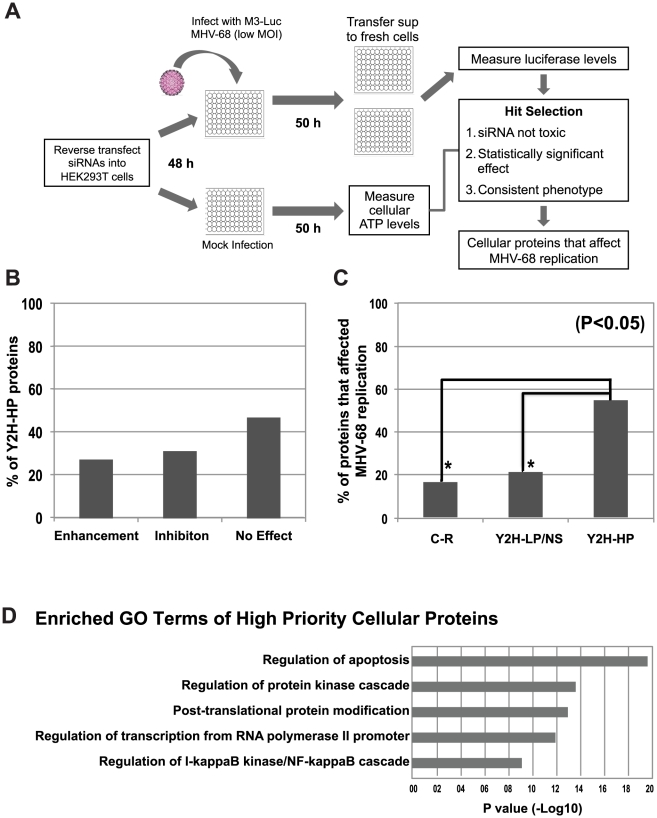
Functional validation of cellular proteins from the MHV-68-cellular protein interaction network. A) Overview of the functional validation approach used to evaluate the effect of cellular interacting proteins on MHV-68 replication. HEK293T cells were reverse transfected with siRNAs targeting cellular genes and infected with M3-Luc MHV-68, which provides a sensitive and conveniently assayed measure of virus replication. The supernatant was collected at 50 and 58 h post-infection and used to infect fresh 293T cells. Luciferase activity was measured at 20 h post-infection. (B) The effect of inhibiting expression of cellular proteins from the Y2H-High priority group (Y2H-HP) on MHV-68 replication. Expression of the 60 highest scoring cellular proteins was inhibited using a single siRNA per gene and MHV-68 replication was assessed as described above. The percentage of siRNAs that enhanced, inhibited, or had no effect on MHV-68 replication is shown. (C) Cellular proteins with high priority scores were more likely to affect MHV-68 replication. Twenty proteins were randomly selected from the Y2H-HP group, the Y2H-low priority and not scored groups (Y2H-LP/NS), and the group of cellular proteins not known to interact with MHV-68 proteins (C-R). The expression of each protein was inhibited with two siRNAs that targeted different regions of the cognate mRNA. MHV-68 replication in the siRNA-transfected cells was measured as described above. Graph shows the percentage of proteins from each group that caused a statistically significant enhancement or inhibition of MHV-68 replication. Asterisks indicate the difference between groups was statistically significant (p<0.05). (D) GO term analysis of high scoring cellular proteins identified in the Y2H screen.

**Figure 6:**
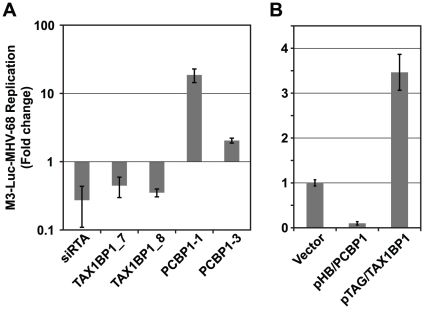
PCBP1 and TAX1BP1 have opposing effects on MHV-68 replication. (A) Effect of siRNAs against PCBP1 and TAX1BP1 on MHV-68 replication. Two distinct siRNAs for each gene were tested for their effect on the replication of M3-Luc-MHV-68 as outlined in Fig. 5. Luciferase values were normalized to the level of replication in cells transfected with the negative control siRNA (siGL3). siRTA is a positive control siRNA against the viral RTA. (B) Effect of over-expressing PCBP1 and TAX1BP1 on MHV-68 replication. HEK293T cells were transfected with parental vector pHB (vector), pHB/PCBP1, or pHB/TAX1BP1 and infected with M3-Luc-MHV-68 as in Fig. 5.

All siRNA experiments were performed in 96-well plates, with each siRNA being transfected into HEK293T cells in triplicate wells (Fig. 5A). As negative controls we used an siRNA with a sequence that did not match any known gene, and siGL3, which targets luciferase (siGL3 will inhibit luciferase RNA expression in the first step, but not the second, and has no effect on MHV-68 replication). As a positive control we used siRTA, which inhibited immediate the MHV-68 early viral gene Replication and Transcription Activator (RTA) and significantly reduced viral replication (Fig. 6A). siRNA-transfected cells were infected with a low titer of the M3-Luc reporter virus (MOI = 0.02) that was empirically determined to enable both positive and negative effects on virus replication to be detected. The impact of the siRNAs on cell viability was assessed in parallel by measuring ATP levels at 50 h post-mock infection, which mimics the condition of the cells at the time of peak virus replication after siRNA treatment (Fig. 5A). siRNAs that reduced cellular ATP levels more than 40% were considered to be toxic and were excluded from further analysis. This experiment was repeated twice with significant correlation in the magnitude of the fold change in viral replication (R = 0.89). More than 50% of the siRNAs that targeted Y2H-HP cellular genes either enhanced or inhibited viral replication (Fig. 5B).

To more rigorously demonstrate the value of this prioritization scheme, we randomly selected 20 genes each from Y2H-HP, Y2H-LP, and the set of human proteins from the reference human PPI network that did not interact with MHV-68 proteins (C-R). Each gene was inhibited with two independent siRNAs using the approach outlined above. Genes for which both siRNAs caused a consistent phenotype, either an enhancement or inhibition of luciferase levels by at least one log_2_ relative to the plate median, were considered to have a significant effect on viral replication (Fig. 5C, S7B, and S8). Overall, we found 53% of the genes in the Y2H-HP group significantly affected MHV-68 replication, whereas only 21% of Y2H-LP and 16% of C-R did so (P<0.05) (Fig. 5C, S7B, and S8). Thus, consistent with our preliminary study (Fig. 5B), cellular proteins with high scores were much more likely to affect MHV-68 replication than cellular proteins with low scores or that were randomly chosen from the reference network. These results suggest that the high scoring cellular proteins (i.e., those that are located near other cellular proteins that also interacted with MHV-68 proteins) are among the most critical cellular targets for the virus, and may reveal regions the virus-cellular protein interaction network that are more likely to affect virus replication.

To determine if particular pathways were over-represented among the Y2H-HP proteins, we repeated the analysis of GO terms for this subset. As shown in Fig. 5D, five major functional clusters were significantly enriched (post-translational protein modification, and regulation of apoptosis, protein kinase cascade, transcription from RNA polymerase II promoter, and I-κB kinase/NF-κB cascade). In contrast, the functional annotations of proteins in the low priority group were mostly involved in regulating cellular organelle organization and biogenesis, lipid homeostasis and DNA biogenesis. Moreover, although some terms, such as regulation of apoptosis, were common to both groups, they were enriched to a greater extent in the high priority group. Given that the Y2H-HP proteins were more likely to affect MHV-68 replication, the fact that these pathways are more highly enriched in the Y2H-HP group suggests that they may be critical for MHV-68 replication. It is possible that low priority proteins may have a more dramatic effect on MHV-68 replication during the infection in *vivo*.

### PCBP1 and TAX1BP have opposing effects on MHV-68 late gene expression

For more in depth follow up experiments we focused on cellular proteins that interacted with a group of viral ORFs that were previously shown to be essential for virus replication in a screen of MHV-68 signature-tagged mutants [19] and that displayed similar phenotypes in subsequent mechanistic studies [45]–[48]. Five viral proteins (ORF18, ORF24, ORF30, ORF31 and ORF34) were involved in regulating late gene expression but were dispensable for early gene expression and DNA replication. In this study we identified TAX1BP1 (Tax1-binding protein) and PCBP1 (Poly(rC)-binding protein 1, also referred to as αCPs and hnRNP E) as potential cellular binding partners of ORF31 and ORF34, respectively. Both proteins were included in the Y2H-HP group and had a significant effect on MHV-68 replication when their expression was inhibited. However, TAX1BP1 and PCBP1 had opposite effects on MHV-68. Whereas inhibiting TAX1BP1 expression reduced M3-Luc-MHV-68 replication, silencing PCBP1 caused an increase (Fig. 6A). We obtained similar results using two additional shRNA constructs for each gene that targeted different sequences on PCBP1 and TAX1BP1, confirming our initial observations. Conversely, when PCBP1 and TAX1BP1 were over-expressed, TAX1BP1 enhanced viral replication ∼3.5-fold, whereas PCBP1 inhibited MHV-68 viral replication approximately 5-fold (Fig. 6B).

**Figure 7:**
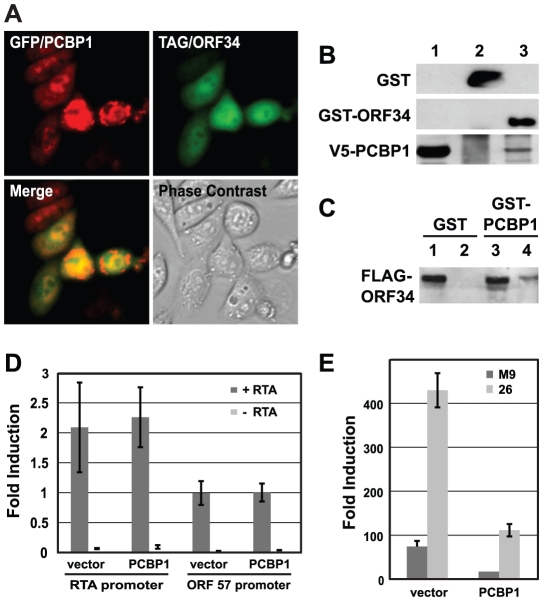
PCBP1 interacts with ORF34 in mammalian cells and negatively regulates late gene expression. (A) Co-localization of PCBP1 and ORF34 in 3T3 cells. Expression plasmids encoding GFP-tagged PCBP1 and FLAG-tagged ORF34 were co-transfected into NIH 3T3 cells. GFP-PCBP1 was visualized by direct fluorescence. FLAG-ORF34 was detected by immunofluorescence assays with anti-FLAG-M2 antibody and Alexa Fluor 594-conjugated anti-mouse IgG antibodies. Panel 1 shows GFP-tagged PCBP1 (green); panel 2, FLAG-ORF34 (red); panel 3, merged image; panel 4, phase contrast image of same field; panel 5, DAPI-stained nuclei. (B) Co-purification of GST-ORF34 and V5 epitope-tagged PCBP1. GST and GST-ORF34 were expressed in BL21 cells and purified with glutathione beads. Extracts from cells that over-expressed V5-PCBP1 were incubated with either GST or GST-ORF34 bound beads. Lane 1, input amount of V5-PCBP1; lane 2, GST pull-down of V5-PCBP1; lane 3, GST-ORF34 pull-down of V5-PCBP1. (C) Co-purification of GST-PCBP1 and FLAG epitope-tagged ORF34. HEK293T cells were transfected with expression plasmids encoding FLAG-ORF34 and either GST or GST-PCBP1 and then infected with MHV-68. Lysate were incubated with glutathione beads and subjected western blot analysis with anti-FLAG antibodies. Lanes 1 and 3, input amount of FLAG-ORF34; lanes 2 and 4, GST and GST-PCBP1 pull-down of FLAG-ORF34, respectively. (D) Over-expression of PCBP1 did not affect expression from early MHV-68 promoters. Reporter constructs encoding the early viral promoters RTA and ORF57 were co-transfected with parental vector pHB/attR (vector) or pHB/PCBP1 plasmid, with or without the pCMV/RTA plasmid. Luciferase activity was measured at 24 h post transfection. (E) Over-expression of PCBP1 inhibited expression from late MHV-68 promoters. Reporter constructs encoding the late viral promoters M9 and ORF26 were co-transfected with parental vector pHB/attR (vector) or pHB/PCBP1 followed by MHV-68 infection at an MOI of 0.5 in 293T cells. Luciferase activity was measured at 24 h post infection.

The interaction of ORF34 and PCBP1 was analyzed further as a model to investigate cellular protein effects on viral replication. We obtained additional support for the physical interaction between ORF34 with PCBP1 by demonstrating binding in GST-pull down assays and co-localization in immuno-fluorescence assays (IFA) in cells (Fig. 7A–C). Since PCBP1 has been shown to affect gene expression at multiple levels via its cis-binding with either DNA or RNA [49]–[51], we hypothesized the interaction of ORF34 and PCBP1 might regulate viral gene expression. A series of viral promoter-linked reporter constructs was used to determine which stage of viral replication was affected by PCBP1. Over-expressing PCBP1 reduced the induction of the luciferase expression from two viral late gene promoters (ORF26 and M9 promoters) upon infection (Fig. 7E). In contrast, the inhibitory effects of PCBP1 were not observed on RTA auto-activation of its own promoter or on RTA-mediated activation of an MHV-68 early gene promoter (ORF57 promoter) (Fig. 7D). This result was consistent with the phenotype of the ORF34 knockout mutant virus, which affected expression of late viral promoter, but did not affect expression at early viral promoters or reduce viral DNA replication [48]. Together, these results suggested that the interaction of ORF34 and PCBP1 regulates viral replication by affecting the transcriptional activities of late promoters.

Since the effect of ORF34 on late gene expression is similar to that of ORF18, ORF24, ORF30 and ORF31, we repeated the GO term analysis of the cellular proteins that interacted with these five viral proteins. Three major functional groups were significantly enriched, including regulation of transcription activities, ubiquitination, and post-translational modification. Together, these results indicate that not only do the viral proteins have similar roles in the viral life cycle, but they also bind to cellular proteins with related functions. Furthermore, it suggests an unexpected role of ubiquitination in the regulation of viral late gene expression. Future studies will focus on the contributions of these pathways to MHV-68 late gene expression.

## Discussion

We report here the first large-scale interactome analysis of MHV-68. Using high-throughput Y2H assays, we created MHV-68 intra-viral and virus-cellular protein interaction networks. In follow-up experiments we demonstrated the quality of the intra-viral protein interaction network by confirming the physical interaction in mammalian cell culture systems. We observed that the cellular proteins that interacted with MHV-68 proteins tended to be closer to one another than expected by chance and exploited this feature to prioritize cellular partners of MHV-68 for in depth analyses. Through gene knockdown experiments we showed that the network neighborhood-based priority score assigned to each cellular factor correlated with the likelihood that the cellular protein affected MHV-68 replication. By integrating experimental screening results with systematic data mining, this study presents a new strategy to analyze and extract biological significance from virus-cellular protein interaction networks.

### The MHV-68 intra-viral protein interaction network

In this study we identified 22 binary interactions between MHV-68 proteins, including 17 interactions not previously identified in any other gamma-herpesvirus (Table S1). Though fewer than reported in other studies [21]–[24], the interactions appear to be of high quality as indicated by greater than 70% confirmation rate of the interactions in co-immunoprecipitation experiments, which compares favorably to confirmation rates of 42 to 53% in previous reports [24], [23]. The reduced number of interactions is likely due to the combination of a stringent Y2H strain (PJ69-4) and a pair of Y2H vectors (pDEST22 and pDEST32) that tend to yield fewer interactions [52].

Despite the stringent selection conditions in our Y2H screens, we identified a number of novel intra-viral protein interactions, several of which involve MHV-68 tegument proteins. Herpesvirus tegument proteins form a complex matrix located between the nuclear capsid and viral envelope and participate in viral assembly and egress [53], [54], [55]. At the center of this sub-network are ORF45 and ORF33, which strongly and reproducibly interact with each other (Fig. S1B). The homologues of ORF45 and ORF33 were also shown to interact with KSHV ORF64, a hub protein that recruits other tegument proteins [22]. Our data suggest that ORF45 may act as a scaffold protein that interacts with tegument proteins ORF33, ORF52, and ORF67, and with the envelope protein ORF68, a homologue of EBV major envelope protein BFLF1 (Fig. S1B). We have previously demonstrated that ORF45 is nearly completely absent from the partially tegumented capsids formed by viruses in which the genes for either ORF52 or ORF33 were disrupted [35], [56] Together, these results suggest that the recruitment of ORF45 to the capsid depends on interactions with both ORF52 and ORF33. The recruitment of ORF45 may further assist the virion assembly by interacting with tegument protein ORF67 and envelope protein ORF68. Further studies are needed to determine if interactions with ORF67 and ORF68 are also required to incorporate ORF45 into capsid or, alternatively, if ORF45 is needed to recruit ORF67 and ORF68.

### The MHV-68-cellular protein interaction network

To identify cellular proteins that interacted with MHV-68 proteins, we screened a well-characterized human liver cDNA library ([36] and unpublished data) and confirmed the initial positives under stringent selection conditions. MHV-68 undergoes productive infection in mouse liver *in vivo* [14]–[16], and, unlike human gamma-herpesvirus, replicates robustly in various human and mouse cell lines in *vitro* ([11]–[13], and unpublished data). Although the liver cDNA library may not include all cellular proteins that interact with MHV-68, liver tissue contains multiple cell types and expresses a broad range of cellular genes. In addition, many more protein-protein interactions have been reported for human proteins than for mouse proteins, which enabled the viral-cellular protein interactions to be placed in the context of the larger cellular protein interaction network. However, one consequence of using a human cDNA library is that most interactions identified in this study involved MHV-68 proteins that are conserved in human herpesviruses. More than half of the interactions that were positive in retest experiments involved 22 MHV-68 proteins that have homologues in all three herpesvirus families and more than 90% involved 32 MHV-68 proteins conserved in gamma-herpesviruses. The six non-conserved MHV68 proteins in our dataset yielded only 20 interactions (8% of the total). For comparison, 27% of the EBV-cellular protein interactions involved EBV-specific proteins [24]. Interactions with murine-specific proteins and proteins important for latency will require additional studies to be identified.

In total, 243 interactions involving 197 cellular proteins were identified and confirmed in our Y2H screens and retest experiments. Among the most enriched features of the cellular proteins revealed by GO enrichment analyses were terms relating to regulation of apoptosis and regulation of NFκB pathway (Fig. S6). This observation is consistent with the notion that viruses encode numerous proteins that counteract cellular immune system and prevent the host cell from prematurely dying [57]–[60]. For example, homologues of the cellular anti-apoptotic protein Bcl-2, including MHV-68 M11 [61], KSHV ORF16 of [62] and EBV BHRF1 [63], are found in all gamma-herpesviruses that inhibit p53-induced apoptosis during infection. Interestingly, one of the prioritized proteins, the coiled-coil myosin-like BCL2-interacting protein (BECN1) that interacts with M11, was independently identified by others to participate in inhibition of apoptosis and autophagy [64], [65]. Another group of proteins, typified by vFLIP of KSHV and LMP1 of EBV, activate NFκB pathway for cell survival by inhibiting early lytic viral gene expression and maintaining viral latency [66]–[73]. However, as shown in herpes simplex virus, the regulation of NFκB pathways might have distinct roles during different stages of viral infection cycles, promoting replication in the lytic phase and regulating host immune responses during latency [74]. In addition to being over-represented in the entire set of interactions, proteins from these pathways ranked highly on the priority list and significantly affected virus replication in siRNA based functional assays, supporting the crucial role of these pathways in MHV-68 replication.

To evaluate the functional roles of the MHV-68-interacting cellular proteins during lytic infection, we used RNA interference and a luciferase-expressing virus. We focused on lytic replication because it is required for the production of viral progeny, is more experimentally accessible than latent infection, and contributes to viral pathogenesis. Tumorigenesis induced by gamma-herpesviruses requires multiple genes expressed during latent as well as lytic replication [10], [75], [76]. Lytic replication is also directly involved in the pathogenesis of diseases such as oral hairy leukoplakia (OHL) caused by EBV and multicentric Castleman disease (MCD) caused by KSHV. Much remains to be understood about the process of lytic replication and the roles of cellular cofactors.

Assay conditions were optimized to reveal both positive and negative effects on MHV-68 replication. From a set of 40 human genes that were targeted with two distinct siRNAs, 13 had a significant effect on virus replication. Of these, six genes caused an increase in MHV-68 replication when their expression was inhibited, whereas seven caused a decrease. It is likely the genes that caused in a decrease in MHV-68 replication when their expression was inhibited encode proteins that are required for virus replication and are being exploited by the virus. In contrast, genes that increased replication when their expression was reduced may either play a negative regulatory role in the virus life cycle or may be part of the cellular antiviral defense.

Among the cellular proteins that caused an increase in MHV-68 lytic replication when their expression was inhibited was PCBP1, which interacted with ORF34. Binding of ORF34 and PCBP1 was confirmed by affinity pull-down experiments and co-localization in mammalian cells, suggesting the interaction occurs *in vivo*. ORF34 was previously implicated in the regulation of late viral gene expression [48]. Consistent with this phenotype, over-expression of PCBP1 inhibited expression from late viral promoters, but had no effect on early viral promoters. Together, the effects of over- and reduced PCBP1 expression on viral replication and promoter activity suggest that PCBP1 plays a negative role in the MHV-68 life cycle. PCBP1 is a multi-functional protein that has been reported to regulate gene expression at the level of transcription, mRNA splicing, mRNA stability, and translation [77]. Multiple viruses, including other gamma-herpesviruses, exploit PCBP1 to regulate of translation, generally in an IRES-dependent manner [78]–[83]. Because no known IRES sequences were present in the promoter constructs, it is unlikely that the effect of PCBP1 on MHV-68 late promoters was at the level of translation. Similarly, since both the early and late viral promoter constructs shared a common 3′ UTR and none encoded splice sites, it is unlikely that PCBP1 altered mRNA stability (which typically occurs through binding to RNA sequences in the 3′ UTR) or splicing of the reporter gene. Rather, we propose that PCBP1 negatively regulated the transcription of late MHV-68 gene. However, it remains to be determined whether the inhibitory role of PCBP1 is by directly binding to the viral DNA or by indirectly modulating cellular gene expression. Regardless of the exact mechanism, these results illustrate the potential for the MHV-68-cellular protein interactome to reveal novel and interesting aspects of virus-cellular protein interactions.

### A network neighborhood approach to prioritize virus-cellular protein interactions

Network analyses of the MHV-68-cellular protein interactome indicated that the cellular proteins that bound to MHV-68 proteins tended to be closer to each other in the reference cellular protein interaction network than expected by chance. We exploited this observation to develop a method to prioritize the cellular proteins from the MHV-68-cellular protein interactome for further characterization. Cellular proteins that interacted with an MHV-68 protein and that were located near other cellular proteins that also bound to MHV-68 proteins were given a positive score, with the magnitude corresponding to the number of MHV-68-interacting proteins located nearby. In essence, this approach identified network neighborhoods that contained multiple cellular proteins targeted by MHV-68. It is important to note that this scoring method is not equivalent to the number of cellular binding proteins (degree) of the MHV-68-interacting proteins. Although the cellular proteins that interacted with MHV-68 proteins participated in more intra-cellular interactions than the average protein in the cellular protein interaction network, the distribution of network distances between MHV-68 interacting proteins was only partially dependent on degree. Similarly, the scoring method is not equivalent to the list of genes bearing enriched GO terms. Although the highly scoring proteins are enriched in several GO terms, particularly those related to cell survival and kinase function, not all proteins with these GO terms received high scores. Thus, the scoring method provides information not obtained from standard network or term enrichment analyses.

Proteins from the MHV-68-cellular protein interactome that scored highly in this scheme were much more likely to affect MHV-68 lytic replication than proteins with a low score or proteins randomly chosen from the cellular protein interaction network that did not interact with MHV-68 proteins (53% vs. 21% or 16%, respectively). A preliminary screen of the 60 highest scoring proteins using a single siRNA per gene yielded comparable results, with slightly more than half the siRNAs causing a significant change in MHV-68 replication. However, the magnitude of the effect on virus replication did not correlate with the score, though this could be due to differences in the effectiveness of the siRNAs in reducing expression of the cellular proteins (Fig. S6). A second caveat is that the scoring system will miss some important proteins in the MHV-68-cellular protein interactome, as indicated by the fact that several low or non-scoring also affected MHV-68 replication. The prioritization approach is also unable to identify cellular proteins that do not bind to MHV-68 but that affect MHV-68 replication indirectly, unless those proteins are located in network neighborhoods target by multiple MHV-68 interactions; genome-wide siRNA screens will be needed to systematically identify cellular cofactors that indirectly impact MHV-68.

Since functionally related proteins cluster together in protein interaction networks [84], [85] an implication of our results is that MHV-68 targets particular cellular processes or pathways through multiple interactions with viral proteins. Consistent with this observation, specific GO terms were enriched in this and other virus-cellular protein interaction networks, which can only occur if multiple functionally related proteins are present. The fact that viruses devote valuable resources to interacting with multiple proteins involved in the same or related functional process suggests that these pathways or process are particularly important for successful replication. However, rather than conferring redundancy to the virus, network neighborhoods that have multiple links to viral proteins appear to represent regions of vulnerability since inhibiting expression of these cellular proteins was more likely to affect virus replication. A similar pattern appears to exist among cellular proteins that bind to HIV-1 proteins [86]. In particular, MacPherson and collaborators recently reported that known HIV-1 host factors are functionally and physically linked clusters within the host cells [86]. Human proteins that interact with HIV-1 can be grouped in a limited number of functionally and physically linked clusters within the host cells [86]. Thus, targeting multiple functionally related proteins may be a general theme in virus-cellular protein interaction networks.

### Comparison of the MHV-68 and EBV-cellular protein interaction networks

In contrast to the screen for virus-virus protein interactions, our screen for cellular proteins that interacted with MHV-68 proteins identified more interactions than a similar study with EBV proteins (243 versus 173) [24]. Although no interactions between homologous viral proteins and the same host factor were found, six cellular proteins were reported in both data sets, which represents a small but statistically significant overlap (p-value = 4.0×10^−3^) [24]. The high false-negative rates of Y2H screens in addition to the use of different versions of the Y2H assay, different cDNA libraries, and different criteria for selecting and confirming true-positive interactions likely contributed to the low overlap. However, this should not be taken to imply that MHV-68 and EBV target completely different sets of cellular proteins, as we expect that direct comparison in pair wise Y2H or co-purification experiments would reveal substantial overlap. Furthermore, our analyses suggest that MHV-68 and EBV target cellular proteins located in the same region of the cellular protein interaction network. The average distance between cellular proteins that interacted with MHV-68 and EBV proteins was significantly closer than the average distance between an equivalently sized set of randomly chosen proteins (MHV-68 to EBV vs. random = 4.67 vs. 5.31, p-value: 3.0×10^−5^). The same comparisons of the average distance between cellular proteins that interacted MHV-68 (or EBV) and influenza virus (Flu) revealed no significant differences compared to randomly chosen cellular proteins (MHV-68 to Flu: 4.98; EBV to Flu; 5.05), suggesting that MHV-68 and EBV target cellular proteins located in the same region of the cellular protein interaction network and that the network proximity of the virus-targeted proteins was specific to herpeviruses [24], [87]. Consistent with this hypothesis, several GO terms, including regulation of apoptosis, I-kappaB kinase/NFκB cascade, and ubiquitin-protein ligase activity, were significantly enriched in both datasets (Fig. S4B). Thus, although the MHV-68 and EBV data sets are undoubtedly incomplete, that MHV-68 and EBV appear to be interacting with similar pathways, but utilizing different proteins to do so. An intriguing implication of this hypothesis is that integrating the gamma-herpesvirus-cellular protein interaction networks may compensate the low coverage of each screen and may reveal important targets missed when the analyses are performed with individual data sets. Indeed, merging the datasets increased the significance of the priority scores of cellular factors that interacted with either MHV-68 or EBV. This effect was much stronger for the smaller of the sets (EBV; Table S4) suggesting the use of composite sets of cellular factors when evaluating newly identified sets of host factors of related viral species may be a valuable approach. A similar strategy has been proposed for evaluating intra-viral protein interaction networks [23].

### Conclusions

Individual herpesviruses have co-evolved with their hosts, adopting diverse strategies to evade the host immune system and to hijack existing cellular machinery [5], [8], [88], [89]. Our study adds to the growing list of gamma-herpesvirus protein-protein interactions and provides additional insight into the complexity of herpesvirus-host cell interactions. The interactions identified here revealed cellular genes that significantly impacted MHV-68 replication and suggest numerous hypotheses about MHV-68 pathogenesis that can be explored in future studies. In addition, we devised a novel strategy to prioritize cellular binding partners of viral proteins for in depth analyses. This nontraditional approach enabled rapid identification of several cellular proteins that negatively or positively affected MHV-68 replication and provides a valuable method for extracting biologically relevant interactions from virus-host protein interaction networks. Finally, this dataset constitutes a valuable resource for comparative studies of the replication strategies of gamma-herpesviruses. As herpesviruses encode a core of conserved genes, such comparative analyses have the potential to reveal cellular proteins and pathways utilized by multiple herpesviruses that may serve as new targets for therapeutic intervention.

## Materials and Methods

### MHV-68 viral ORF library

MHV-68 open reading frames (ORFs) were PCR amplified from the MHV-68 viral DNA and cloned into pENTR vectors by using Gateway pENTR/D/TOPO cloning kit (Invitrogen). Total of 84 MHV-68 ORF pENTR clones, including 64 full length and 14 fragmented viral ORF clones for ORF larger than 1.5 kb, and 6 ORF clones without trans-membrane domain, were generated. Inserts in entry clones were sequence verified and transferred to destination vectors for expression in yeast and mammalian system using Gateway LR Clonase enzyme mix (Invitrogen) suitable for N-terminus tagging. Destination clones, pTAG and pHB vectors, were constructed by adding FLAG/Calmodulin Binding Peptide (CBP) and V5/HIS fusion tag respectively for co-immunoprecipitation (Co-IP). pYFP and pRFP vectors were constructed by adding the genes encoding yellow fluorescent protein and *Discosoma sp.* red fluorescent protein (DsRed) respectively for immunofluorescence assay (IFA) . Recombination of inserts from pENTR clones results in an in frame fusion at the 3′ end of the fusion epitope tag or fluorescent tag.

### Yeast-two-hybrid screens

#### Strains, plasmids and libraries

*Saccharomyces cerevisiae* strains used in this study are listed in Table S5. Yeast were maintained using standard laboratory procedures [90]. To test for interactions between viral proteins, MHV-68 ORFs and ORF fragments were transferred into pDEST22 (*GAL4* activation domain, *TRP1*) and pDEST32 (*GAL4* DNA binding domain, *LEU2*) using the Gateway recombination system (Invitrogen) and transformed into the yeast strains PJ69-4a and PJ69-4α, respectively. To identify human proteins that interacted with viral proteins, MHV-68 ORFs and ORF fragments were recombined into the Gateway vector pXDGATcy86 (*GAL4* DNA binding domain, *TRP1*) [91] and transformed into the yeast strain R2HMet. Viral DNA binding domain clones were used to screen a human liver cDNA library generated in plasmid pOAD.103 (*GAL4* activation domain, *LEU2*, *URA3* to select for fragments cloned in frame with *GAL4*) [36]. The cDNA library contained 9.6×10^5^ independent clones with an average insert size of 840 bp.

### Self-activation test

DNA-binding domain strains were mated to PJ69-4a cells transformed with the parental activation domain plasmid pDEST32. Diploid yeast were selected on synthetic drop-out medium (SD) lacking tryptophan and leucine supplemented with 0.003% adenine (SD–LEU–TRP+ADE) and plated on SD medium lacking leucine, tryptophan, and histidine (SD–LEU–TRP–HIS) and containing 0, 1, 3, 5, 10, 20, 50, 75, 100, 125, and 150 mM 3-amino-1,2,4-triazole (3-AT, a competitive inhibitor of His3). Y2H screens were performed using the lowest concentration of 3-AT that suppressed yeast growth in the absence of an interacting activation domain fusion (1 or 3 mM for most DNA-binding domain constructs).

### Y2H screens for interactions between MHV-68 proteins

PJ69-4a cells expressing MHV-68 activation domain fusions were arrayed in 96-spot format on SD–LEU–TRP+ADE medium and converted to 384-spot format using a Biomek FX robot (Beckman Coulter, Brea, CA) equipped with 96 pin replicating head; each strain was represented four times in the 384-spot format. Y2H assays were performed by replica pinning the activation domain clones to YPDA (yeast extract-peptone-dextrose plus 0.003% adenine) medium and then pinning a single DNA-binding-domain clone on each spot. Diploid yeast containing the DNA-binding domain and activation domain plasmids were selected on SD–LEU–TRP+ADE and replica-pinned onto two independent Y2H selection media: (i) SD–LEU–TRP-HIS supplemented with the optimal 3-AT concentration as determined above; and (ii) SD–LEU–TRP−ADE. Yeast growth was assessed after 7 to 14 days at 30 C. Pairs of activation and DNA-binding domain fusions that stimulated yeast growth on both Y2H selection media in at least three spots were judged to be true positives.

### Y2H library screens

Y2H screens were performed by mating as described by [92] with the following modifications: Mid log phase R2HMet cells (1×10^7^ cfu) containing a pXDGATcy86 clone were mixed with 5×10^6^ cfu of the human liver activation domain library in YPD (yeast extract-peptone-dextrose medium), pH 3.5, and incubated 1 h with rotation, at which point the media was replaced with 3 ml of YPDA, pH 4.5. The cells were collected by centrifugation and incubated overnight at room temperature to allow mating. Mating efficiency was estimated by plating an aliquot of each screen on SD–LEU–TRP+ADE, which selected for diploid yeast containing both Y2H plasmids; typical yields were 3–5×10^5^ diploids per screen. The remaining yeast were collected by centrifugation, washed once with dH2O, and plated on SD–LEU–TRP-HIS supplemented with the optimal 3-AT concentration as determined above. Plates were incubated at 30°C until colonies appeared (4 to 10 days). All DNA-binding domain clones were screened at least twice. Yeast colonies that grew on SD–LEU–TRP-HIS+3-AT (up to 48 per screen) were picked and grown overnight in liquid YPDA medium. The human gene insert in pOAD.103 was then PCR-amplified with primers 5′-CGACGACGAGGATACGCCACCGAAC-3′, and 5′-GAGCTTCGCAGCAACCGGACTAGGA-3′ and sequenced from the 5′ end with primer 5′-ATACGCCACCGAACCCTAAGAAA-3′. The gene identity was assigned by querying the human RefSeq database (downloaded 3/4/08) using Cross Match.

### Confirmation of Y2H interactions

PCR products of activation domain inserts from unique interactions identified in the Y2H library screens were re-cloned into pOAD.103 by in vivo homologous recombination in the yeast strain BK100 [93]. Two independent activation domain inserts were re-cloned for interactions that were identified more than once in the library screens. Plasmids were verified by PCR and sequencing. Yeast containing the remade activation domain clones were spotted on solid SD–LEU–TRP+ADE medium in 96-spot format with a BioMek FX robot. Each spot was then transferred to solid SD–LEU–URA+ADE medium in quadruplicate to create a 384-spot array. Strains expressing the DNA-binding domain fusions were grown to mid log phase in SD–TRP+ADE medium, collected by centrifugation, resuspended in fresh media and pinned onto solid YPDA medium in 384-spot format corresponding to the activation domain clone array described above. The activation domain array was then replica pinned onto the same plates. After 2 days at 30°C, yeast were replica pinned onto solid SD–LEU–TRP+ADE medium to select for diploid yeast. Plates were incubated 3 days at 30°C and the yeast replica pinned onto three plates: (i) SD–LEU–URA–TRP-HIS containing the minimum concentration of 3-AT to suppress background growth; (ii) SD–LEU–URA–TRP–HIS containing 3-AT at a concentration one step above minimum level required to suppress background growth; and (iii) SD–LEU–URA–TRP–ADE. Plates were incubated 7 days at 30 C and imaged at days 3 and 7. Interactions were scored as positive if at least 3 of the 4 spots for each interaction displayed growth above background growth of in-plate negative controls on both media lacking HIS and ADE.

### Cell lines and antibodies

Human embryonic kidney (HEK)-293T (293T) cells and NIH/3T3 (3T3) cell lines were both maintained at 37 °C in 5% CO2 atmosphere in Dulbecco's modified Eagle's medium supplemented with 10% fetal bovine serum (293T) or bovine calf serum (3T3) and 1% penicillin and streptomycin. Anti-FLAG-M2 was purchased from Sigma. Anti-V5, secondary fluorescent antibodies (Alexa Fluor 594 goat anti-mouse IgG, Alexa Fluor 488 goat anti-mouse IgG) were purchased from Invitrogen.

### Physical and functional validation of the PPIs

#### Co-immunoprecipitation and affinity pull-down assays

Co-immunoprecipitation experiments were performed with FLAG and V5 epitope-tagged proteins. 293T cells at 70–80% confluence were transfected with plasmids encoding FLAG (pTAG) and V5 (pHB) epitope-tagged cDNAs using Lipofectamine with Plus reagents (Invitrogen, Carlsbad, CA) according to manufacturer's instructions. The cells were infected with MHV-68 (MOI = 0.5) at 24 hrs post-transfection and lysed at 18 hrs post-infection with radiomimmunoprecipitation (RIPA) Buffer (50 mM Tris-HCl pH 7.4, 0.5% NP-40, 150 mM NaCl, 1 mM EDTA) plus Complete Protease Inhibitor Cocktail Tablets (Roche). Protein G-sepharose beads (GE Healthcare) were washed once with RIPA buffer and incubated with 1 µg anti-FLAG (Sigma Aldrich), anti-V5 (Invitrogen) or anti-mouse IgG antibodies as negative control (Santa Cruz Biotechnology) overnight at 4°C. Cell lysates were sonicated, mixed with antibody-G-sepharose beads in RIPA buffer plus protease inhibitors for 4 hrs at 4°C with constant shaking. Beads were washed three times with RIPA buffer and resuspended with 50 µl of SDS-PAGE sample buffer.

For affinity pull-down assay, viral ORFs were transferred into the Gateway destination plasmid pDEST15 (Invitrogen, Carlsbad, CA), in which the glutathione S-transferase (GST) gene is fused to the 5′ end of the insert. GST fusion viral proteins were expressed in *E. coli* BL21 cells and purified with glutathione agarose beads (Invitrogen) by standard methods. HEK293T cells were transfected with cellular cDNA clones (pHB) fused with V5 epitope-tagged and lysed in RIPA buffer. Cell extracts were incubated with GST only or GST-Viral ORF-bound beads for 1 h at room temperature. Beads were washed three times with RIPA buffer and resuspended with 50 µl of SDS-PAGE sample buffer. The samples were subjected to SDS-PAGE and western blotting as described below.

### Western blotting

Protein samples were resolved by SDS-PAGE on 12.5% or 15% polyacrylamide gels and transferred to polyvinylidene fluoride membrane. Blots were blocked overnight in 5% nonfat dried milk in phosphate-buffered paline (PBS) with 0.2% Tween 20 (PBST) and probed with anti-V5 (1∶5000) or anti-FLAG-M2 (1∶5000) followed by horseradish peroxidase (HRP) conjugated rabbit anti-mouse IgG secondary antibody (1∶5000) or with HRP conjugated primary antibody, anti-Flag-HRP (1∶5000) and anti-V5-HRP (1∶5000). Blots were imaged using the ECL detection kit (GE Healthcare) according to manufacturer's instructions.

### Co-localization and immunofluorescence assays

Corresponding FLAG (pTAG), V5 (pHB) tagged, GFP, YPF, or RFP cDNA expression clones for protein-protein interactions of interest were co-transfected into 20 million 3T3 cells by using BioT reagent according to manufacturer's instructions in 96 wells plate. At 24–28 hours post-transfection, cells were washed with 100 µl of cold Phosphate-buffered Saline (PBS) and fixed with 50 µl cold methanol on ice for 15 minutes. Fixative was aspirated and cells were washed with 150 µl cold PBS four times and blocked with 100 µl blocking buffer (1× PBS, 10% Fetal Bovine Serum, 1% Bovine Serum Albumin and 0.1% Triton-X 100) for 30 minutes at room temperature. Fifty µl of diluted primary antibody was added to each well and incubated for one hour at room temperature. Anti-FLAG-M2 antibodies and anti-V5 antibodies were used for detection of pTAG and pHB expression clones. Cells were washed with cold PBS four times and incubated with 50 µl of appropriate fluorescent-conjugated secondary antibody for another hour. DAPI (Invitrogen, Carlsbad, CA) were used for nuclease stain for 15 minutes at room temperature.

### siRNA knockdown and cDNA over-expression experiments

Predesigned siRNAs were purchased from Qiagen. The siRNA sequences were designed by Qiagen using BioPredsi neural-network based on a large number of siRNA datasets, which has been shown to have about 70% knockdown efficiency on targeted transcripts [94]–[97]. SiGL3 and Negative Control siRNA were used as controls (Qiagen). Cellular cDNA expression clones were PCR amplified from revered-transcribed cDNA from 293T and cloned into Gateway system as previous described. Two pmol of siRNA or 200 ng of cDNA expression clones (pTAG or pHB) were reverse-transfected into 293T cells with Lipofectamine 2000 (Invitrogen) according to manufacturer's instructions in 96 well format and infected with M3-Luc-MHV-68 at 48 h (siRNA) or 24 h (cDNA) post-transfection. The M3-Luc MHV-68 reporter virus was constructed by inserting the firefly luciferase gene downstream of the lytic gene M3 promoter (constructed by Dr. Seungmin Hwang). Fifty µl of supernatant containing infectious viral particles from infected cells was carefully transferred to fresh 293T cells 50 h post-infection. Cell extract for luciferase activity assay was prepared by adding 60 µl of Bright-glo buffer (Promega) to each well 20 h post-transfer. Transfections were performed in triplicate and duplicate readings of luciferase activity were obtained (Molecular Devices). Cytotoxicity assays were performed on siRNA-transfected cells that were mock-infected at 48 h post-siRNA transfection. ATP levels were measured at 50 h post-mock infection with ATPLite Luminescence ATP Detection Assay System (PerkinElmer) according to the manufacturer's protocol for a 96-well microplate.

To be considered for this analysis, we required that the siRNA treatment resulted in at least 60% of ATP level compare to the negative controls and that the measurements had less than 30% of the standard deviation (SD) within the triplicate of each siRNA treatments. The average signals of the triplicate non-specific siRNA treatments were similar to the average to the median of each plate. To avoid plate-to-plate variation, the averages of luciferase activities of the triplicate measurements were normalized to the median of all the readings on the same plate which is close to the average of the negative control. Treatments that increased the luciferase levels to ≥the median plus two times the standard deviation within triplicates of negative controls, which corresponds to plus one Log_2_ fold, were considered to have enhanced MHV-68 replication. Similarly, treatments that reduced luciferase levels to ≤the median minus two times the standard deviation within triplicates of negative controls, which corresponds to minus one Log_2_ fold, were considered to have inhibited virus replication.

### Viral promoter assays

Viral promoter reporter plasmids were constructed in which the firefly luciferase gene was linked to either early (M3 and ORF57) or late (M9 and ORF26) viral gene promoters. HEK293T cells were transfected with early viral promoter-firefly luciferase reporter plasmids with cDNA expression construct (pHB/PCBP1) or vector alone (pHB/attR) with/without pCMV/RTA co-transfection. Late viral promoter-firefly luciferase reporter plasmids were co-transfected with cDNA expression construct (pHB/PCBP1) or vector alone (pHB/attR) followed by MHV-68 (MOI:0.5) infection at 24 h post-transfection in 293T cells. A CMV-driven *Renilla* luciferase plasmid was co-transfected with all of the transfection combinations to serve as a control for transfection efficiency. Cells were lysed with 100 µl Passive Lysis Buffer (Promega) at 24 h post-transfection for early promoters assays, and at 24 h post-infection for late viral promoter assays. Lysates were assayed for firefly and Renilla luciferase activity using Dual-Luciferase Reporter assay kit (Promega) flowing the manufacture protocol.

### Cellular protein interaction network

The reference protein-protein interaction network was constructed by merging binary interactions reported in DIP [39], IntAct [98] and MINT [99] databases; common curation rules adopted by these databases within IMEx Consortium [100] ensure consistent annotation of the experiments and similar level of quality control. Only direct and physical interactions supported by at least one small-scale experiment, defined operationally as these reported in a paper describing less than 100 independent experiments were included. Binary, physical interactions inferred from the multi-protein complexes according to the spoke model (i.e. bait-prey pairs) were included only for complexes composed of less than 30 proteins. Alternative forms of the proteins coded by the same gene were merged into one node within the network. The relatively stringent criteria used here resulted in a reference human protein-protein interaction network composed of 4280 proteins connected by 8939 interactions. Whereas of only moderate size when compared to interaction networks that include the results of large-scale interaction screens and computational predictions, the reference network constructed here represents a subset of the currently known human interactome that is supported by a wide array of independent experiments reported in more than 3,200 publications. It thus attempts to minimize experimental and computational bias possible when constructing interaction sets based predominantly on the computationally processed results of a small number of high throughput experiments and computational protein interaction predictions.

Distances between two proteins within the reference interaction network were calculated as the length of the shortest path connecting the corresponding network vertices with each edge of the network assigned a weight of 1. Disconnected pairs of proteins were excluded from the distance analysis.

The distribution of the pair-wise network distances, calculated as the length of the shortest path between the cellular proteins identified in the Y2H screen, was constructed by taking into consideration every protein pair connected within the reference network described above. It was compared to the distribution of distances between equivalent numbers of proteins randomly selected from the reference network. Statistical significance of the differences in the distribution shape was evaluated by Monte-Carlo method. To this end distribution of the chi-square statistic:
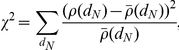
where ρ(d_N_) is the frequency of observing d_N_ distance, was calculated for 10^4^–10^5^ random sets of cellular factors and subsequently to used to estimate p-value as p(C^2^>C^2^_obs_).

### Network-based priority score

In order to prioritize cellular factors initially identified in the Y2H screen, a simple score was calculated by counting, for each cellular factor, the number of other cellular factors located not farther than four interactions away within the reference human protein interaction network (see Fig. 4A). The significance of the priority scores was estimated by Monte-Carlo method as described above.

### Interaction network and GO terms analysis

All the interactomes were generated by an open sources plateform for network analysis and visualization software, Cytoscape (http://cytoscape.org/index.php). GO terms analysis generated using the BinGO [101] plugin within Cytoscape. The functional annotations of cellular proteins were analyzed together with their immediate neighbors in the cellular protein inteaction reference network under the GO background of *Homo Sapiens*. GO terms with specific functional annotation with corrected p- value less than 1×10^−3^ were selected.

### Interaction data deposition

The set of interactions reported in this paper has been submitted directly to the DIP database and assigned IMEx Consortium (http://www.imexconsortium.org) IM-15822 accession number.

## Supporting Information

Figure S1 Confirmation of MHV-68 intra-viral protein-protein interactions. (A) Interaction partners from the Y2H screen were cloned into destination vectors with either a FLAG or V5 epitope tag and co-transfected into 293T cells. Over-expressed FLAG and V5 tagged viral protein lysates were immunoprecipitated with anti-FLAG, anti-V5, or anti-mouse-IgG (negative control) antibodies. Eluates from the co-immunoprecipitations were subjected to SDS-PAGE and western blotting with anti-FLAG-HRP (upper panel) and anti-V5-HRP (bottom panel) antibodies. A summary of results from the co-IP experiments is shown in Table S1. (B) MHV-68 tegument protein interactions. The colors and shapes of viral proteins and intra-viral interactions are shown as described in legend to Fig. 2. Interactions between viral homologues identified by Rozen et al., 2008 [24] are shown in red.
(EPS)

Figure S2 Combined gamma-herpesvirus intraviral protein interaction network. Orthologous proteins from MHV-68, EBV, and KSHV were grouped together and their intra-viral interactions indicated by different colored lines. Interactions between EBV and KSHV proteins mostly clustered on opposite sides of the network. Different orthologous proteins from EBV and KSHV were involved interactions, which accounts for the low number of interactions that are shared. The most likely explanation is that, despite their sequence similarity, different subsets of proteins from EBV and KSHV were functional in the yeast two-hybrid assay.
(EPS)

Figure S3 The MHV-68-cellular protein interaction network. Lines indicate protein-protein interactions. Arrows point from baits to preys. Interactions between cellular proteins are not shown. Viral proteins are shown as a ring and are color coded according to their putative functions in the MHV-68 life cycle. Circles represent nonessential MHV-68 proteins. Rectangles and circles are colored to indicate protein functions (blue, DNA replication complex; red, regulatory proteins; orange, envelope protein; yellow, capsid; magenta, assembly or egress; white, unknown function). Rectangles designate viral proteins essential for MHV-68 lytic replication. Cellular proteins are shown as circles and labeled with their official gene symbol. Cellular proteins inside the ring of viral proteins interact with more than one viral protein, whereas those outside the ring interact with a single MHV-68 protein. Cellular proteins are color-coded according to their network neighborhood-based priority score. Green indicates high priority score, yellow indicates low priority score, and white indicates no priority score. The size of the circle indicates the magnitude of the effect on MHV-68 lytic replication caused by inhibiting the expression of the cellular gene by RNAi. Larger circle implies greater effect; small circle, no effect or not tested. Font color of the cellular protein label indicates the direction of the effect on MHV-68 lytic replication (red, inhibiting expression of the cellular gene increased replication, dark green: inhibiting expression of the cellular gene decreased replication, black, no effect or not tested).
(EPS)

Figure S4 Enriched GO terms of cellular proteins that interacted with MHV-68 or EBV proteins [24]. (A) MHV-68 proteins were categorized according to their putative functions (replication complex (1), regulation (2), assembly/egress (3), and unknown). Bar graphs show the −Log_10_ (p-value) of the enriched GO terms of the cellular proteins, plus their immediate neighbors in reference cellular protein interaction network, that interacted with the different groups of MHV-68 proteins. Enrichment analysis was performed using the Cytoscape plugin BINGO. (B) GO term analysis of MHV-68 and EBV interacting cellular proteins. GO term enrichment was performed as in part (A). Terms with a pvalue<1×10^−3^ are shown. The thicknesses of the line connecting the GO terms to the virus indicates the significance (−Log_10_ (p-value)).
(EPS)

Figure S5 Network context of the cellular proteins identified in genome-wide Y2H screen. (A) The distribution of the network distances between cellular proteins identified in the Y2H screen was not affected by the removal of cellular proteins targeted by more than one viral protein. Solid line, distance distribution for the complete set of MHV-68-interacting proteins; dashed line, distance distribution between after removing cellular proteins that interacted with more than one MHV-68 protein; (B) The distribution of network distances between proteins randomly selected from the reference network was not affected by their degree. Closed circles show the average distance distribution of randomly chosen sets of cellular proteins; the degree distribution of this group reflected that found in the reference cellular protein interaction network. Open circles indicate the average distance distribution of randomly chosen sets of cellular proteins with a modified degree distribution that matched with the MHV-68 data set. Data sets from both cases were equivalent in size the set of MHV-68-interacting cellular proteins.
(EPS)

Figure S6 Distribution of network distances between cellular proteins that interacted with EBV proteins. The distances between cellular proteins that interacted with EBV proteins in the reference cellular protein interaction network [24] (i.e., the number of protein-protein interactions that must be passed through to connect a pair of proteins) were calculated and plotted. Closed circles (thick line) represent the observed distribution of distances between the MHV-68-interacting proteins. Bars (thin line) show the average distance distribution from an equivalent number of randomly selected cellular proteins.
(EPS)

Figure S7 Effect of inhibiting expression of cellular proteins from the MHV-68-cellular protein interaction network on MHV-68 replication. (A) Luciferase activity from cells infected with M3-Luc-MHV-68 directly correlates with virus titer. Increasing amounts of M3-Luc-MHV-68 were added to 293T cells and luciferase activity was assayed from infected cell lysates at 20 h post infection. The average luciferase value (relative light units) of triplicate infections is shown and plotted against plaque forming units determined by plague assays. (B) Summary of the effect of inhibiting expression of cellular proteins from high priority (Y2H-HP) and low priority/no score (Y2H-LP/NS) groups on virus replication (shown in Fig. 5C). The graph show the percentage of proteins in which from each group that enhanced (white bars), inhibited (black bars), or had no effect (gray bars) on M3-Luc-MHV-68 replication.
(EPS)

Figure S8 MHV-68 replication in cells transfected with siRNAs targeting cellular genes. Twenty proteins were randomly selected from the high priority (Y2H-HP) group, the low priority/no score group (Y2H-LP/NS), and the set of cellular proteins (C-R) not known to interact with MHV-68. Two siRNAs targeting each gene encoding these proteins were independently transfected into 293T cells in 96-well plates in triplicate. Replication of M3-Luc-MHV-68 was then measured as described in Fig. 5 and the Materials and Methods. Graphs show the fold change (log_2_ scale) in M3-Luc-MHV-68 replication in siRNA-treated cells relative to cells treated with a nonspecific siRNA. Data was normalized to the plate median and negative controls. Box indicates siRNAs targeting a single gene in which both siRNAs had a consistent and statistically significant effect on MHV-68 replication (either enhancement or inhibition). Underline indicates siRNAs that reduced cell viability. MHV-68 replication was not reported for these siRNAs due to the confounding effects of cellular toxicity.
(EPS)

Table S1 MHV-68 intra-viral protein-protein interactions.
(PDF)

Table S2 MHV-68 virus-cellular protein-protein interactions.
(PDF)

Table S3 Priority rank, priority score, protein degree, and effect on MHV-68 replication of cellular proteins from the MHV-68-cellular protein interaction network. The effects of silencing expression of cellular proteins (Fig. S6C) on MHV-68 replication were annotated as following: enhanced MHV-68 replication (+), inhibited MHV-68 replication (−), cellular toxicity (toxic).
(PDF)

Table S4 Priority rank of cellular proteins based on the merged EBV- [24] and MHV-68-PPI hit list.
(PDF)

Table S5 Yeast strains used in this study.
(PDF)

## References

[1] Pellett PE, Roizman B, Knipe DM, Howley PM (2001). The family herpesviridae: a brief introduction.. Fields Virology. 4th ed.

[2] Renne R, Lagunoff M, Zhong W, Ganem D (1996). The size and conformation of Kaposi's sarcoma-associated herpesvirus (human herpesvirus 8) DNA in infected cells and virions.. J Virol.

[3] Babcock GJ, Decker LL, Volk M, Thorley-Lawson DA (1998). EBV persistence in memory B cells in vivo.. Immunity.

[4] Speck SH, Ganem D (2010). Viral latency and its regulation: lessons from the gamma-herpesviruses.. Cell Host Microbe.

[5] Rickinson A, Kieff E, Fields BN, Knipe DM, Howley PM (2007). Epstein-Barr Virus and its replication.. Fields Virology.

[6] Chang Y, Cesarman E, Pessin MS, Lee F, Culpepper J (1994). Identification of herpesvirus-like DNA sequences in AIDS-associated Kaposi's sarcoma.. Science.

[7] Epstein MA, Achong BG, Barr YM (1964). Virus Particles in Cultured Lymphoblasts from Burkitt's Lymphoma.. Lancet.

[8] Rezk SA, Weiss LM (2007). Epstein-Barr virus-associated lymphoproliferative disorders.. Hum Pathol.

[9] Grundhoff A, Ganem D (2004). Inefficient establishment of KSHV latency suggests an additional role for continued lytic replication in Kaposi sarcoma pathogenesis.. J Clin Invest.

[10] Kenney S (2006). Theodore E. Woodward Award: development of novel, EBV-targeted therapies for EBV-positive tumors.. Trans Am Clin Climatol Assoc.

[11] Usherwood EJ, Stewart JP, Nash AA (1996). Characterization of tumor cell lines derived from murine gammaherpesvirus-68-infected mice.. J Virol.

[12] Virgin HW, Latreille P, Wamsley P, Hallsworth K, Weck KE (1997). Complete sequence and genomic analysis of murine gammaherpesvirus 68.. J Virol.

[13] Simas JP, Efstathiou S (1998). Murine gammaherpesvirus 68: a model for the study of gammaherpesvirus pathogenesis.. Trends Microbiol.

[14] Hwang S, Wu T-T, Tong LM, Kim KS, Martinez-Guzman D (2008). Persistent gammaherpesvirus replication and dynamic interaction with the host in vivo.. J Virol.

[15] Sunil-Chandra NP, Efstathiou S, Nash AA (1992). Murine gammaherpesvirus 68 establishes a latent infection in mouse B lymphocytes in vivo.. J Gen Virol.

[16] Virgin HW, Speck SH (1999). Unraveling immunity to gamma-herpesviruses: a new model for understanding the role of immunity in chronic virus infection.. Curr Opin Immunol.

[17] Martinez-Guzman D, Rickabaugh T, Wu T-T, Brown H, Cole S (2003). Transcription Program of Murine Gammaherpesvirus 68.. J Virol.

[18] Bortz E, Whitelegge JP, Jia Q, Zhou ZH, Stewart JP (2003). Identification of proteins associated with murine gammaherpesvirus 68 virions.. J Virol.

[19] Song MJ, Hwang S, Wong WH, Wu T-T, Lee S (2005). Identification of viral genes essential for replication of murine gamma-herpesvirus 68 using signature-tagged mutagenesis.. Proc Natl Acad Sci U S A.

[20] Arumugaswami V, Sitapara R, Hwang S, Song MJ, Ho TN (2009). High-resolution functional profiling of a gammaherpesvirus RTA locus in the context of the viral genome.. J Virol.

[21] Uetz P, Dong Y-A, Zeretzke C, Atzler C, Baiker A (2006). Herpesviral protein networks and their interaction with the human proteome.. Science.

[22] Rozen R, Sathish N, Li Y, Yuan Y (2008). Virion-Wide Protein Interactions of Kaposi's Sarcoma-Associated Herpesvirus.. J Virol.

[23] Fossum E, Friedel CC, Rajagopala SV, Titz B, Baiker A (2009). Evolutionarily Conserved Herpesviral Protein Interaction Networks.. PLoS Pathog.

[24] Calderwood MA, Venkatesan K, Xing L, Chase MR, Vazquez A (2007). Epstein-Barr virus and virus human protein interaction maps.. Proc Natl Acad Sci U S A.

[25] Stellberger T, Häuser R, Baiker A, Pothineni VR, Haas J (2010). Improving the yeast two-hybrid system with permutated fusions proteins: the Varicella Zoster Virus interactome.. Proteome Sci.

[26] Speck SH, Virgin HW (1999). Host and viral genetics of chronic infection: a mouse model of gamma-herpesvirus pathogenesis.. Curr Opin Microbiol.

[27] Stelzl U, Worm U, Lalowski M, Haenig C, Brembeck FH (2005). A human protein-protein interaction network: a resource for annotating the proteome.. Cell.

[28] Uetz P, Giot L, Cagney G, Mansfield TA, Judson RS (2000). A comprehensive analysis of protein-protein interactions in Saccharomyces cerevisiae.. Nature.

[29] Yokoyama N, Fujii K, Hirata M, Tamai K, Kiyono T (1999). Assembly of the epstein-barr virus BBLF4, BSLF1 and BBLF2/3 proteins and their interactive properties.. J Gen Virol.

[30] Constantin N, Dodson MS (1999). Two-hybrid analysis of the interaction between the UL52 and UL8 subunits of the herpes simplex virus type 1 helicase-primase.. J Gen Virol.

[31] McLean GW, Abbotts AP, Parry ME, Marsden HS, Stow ND (1994). The herpes simplex virus type 1 origin-binding protein interacts specifically with the viral UL8 protein.. J Gen Virol.

[32] Barnard EC, Brown G, Stow ND (1997). Deletion mutants of the herpes simplex virus type 1 UL8 protein: effect on DNA synthesis and ability to interact with and influence the intracellular localization of the UL5 and UL52 proteins.. Virology.

[33] Biswas N, Weller SK (1999). A mutation in the C-terminal putative Zn2+ finger motif of UL52 severely affects the biochemical activities of the HSV-1 helicase-primase subcomplex.. J Biol Chem.

[34] Zhu FX, Yuan Y (2003). The ORF45 protein of Kaposi's sarcoma-associated herpesvirus is associated with purified virions.. J Virol.

[35] Guo H, Wang L, Peng L, Zhou ZH, Deng H (2009). Open reading frame 33 of a gammaherpesvirus encodes a tegument protein essential for virion morphogenesis and egress.. J Virol.

[36] Vignali M, McKinlay A, LaCount DJ, Chettier R, Bell R (2008). Interaction of an atypical Plasmodium falciparum ETRAMP with human apolipoproteins.. Malar J.

[37] LaCount DJ, Vignali M, Chettier R, Phansalkar A, Bell R (2005). A protein interaction network of the malaria parasite Plasmodium falciparum.. Nature.

[38] Vidalain P-O, Boxem M, Ge H, Li S, Vidal M (2004). Increasing specificity in high-throughput yeast two-hybrid experiments.. Methods.

[39] Salwinski L, Miller CS, Smith AJ, Bowie JU, Eisenberg D (2004). The Database of Interacting Proteins: 2004 Update.. Nucleic Acids Res.

[40] Aranda B, Achuthan P, Alam-Faruque Y, Armean I, Bridge A (2010). The IntAct molecular interaction database in 2010.. Nucleic Acids Res.

[41] Ceol A, Chatr Aryamontri A, Licata L, Peluso D, Briganti L (2010). MINT, the molecular interaction database: 2009 update.. Nucleic Acids Res.

[42] Yook S-H, Oltvai ZN, Barabási A-L (2004). Functional and topological characterization of protein interaction networks.. Proteomics.

[43] Huang J-Y (2009). Tomography of functional organization in protein–protein interaction network.. Physica A.

[44] Li X, Feng J, Chen S, Peng L, He W-W (2010). Tpl2/AP-1 enhances murine gammaherpesvirus 68 lytic replication.. J Virol.

[45] Jia Q, Wu T-T, Liao H-I, Chernishof V, Sun R (2004). Murine gammaherpesvirus 68 open reading frame 31 is required for viral replication.. J Virol.

[46] Arumugaswami V, Wu T-T, Martinez-Guzman D, Jia Q, Deng H (2006). ORF18 is a transfactor that is essential for late gene transcription of a gammaherpesvirus.. J Virol.

[47] Wong E, Wu T-T, Reyes N, Deng H, Sun R (2007). Murine gammaherpesvirus 68 open reading frame 24 is required for late gene expression after DNA replication.. J Virol.

[48] Wu T-T, Park T, Kim H, Tran T, Tong L (2009). ORF30 and ORF34 are essential for expression of late genes in murine gammaherpesvirus 68.. J Virol.

[49] Huo L-R, Zhong N (2008). Identification of transcripts and translatants targeted by overexpressed PCBP1. Biochim.. Biophys Acta.

[50] Leffers H, Dejgaard K, Celis JE (1995). Characterisation of two major cellular poly(rC)-binding human proteins, each containing three K-homologous (KH) domains.. Eur J Biochem.

[51] Mili S, Shu HJ, Zhao Y, Piñol-Roma S (2001). Distinct RNP complexes of shuttling hnRNP proteins with pre-mRNA and mRNA: candidate intermediates in formation and export of mRNA.. Mol Cell Biol.

[52] Rajagopala SV, Hughes KT, Uetz P (2009). Benchmarking yeast two-hybrid systems using the interactions of bacterial motility proteins.. Proteomics.

[53] Mettenleiter TC, Klupp BG, Granzow H (2009). Herpesvirus assembly: An update.. Virus Research.

[54] Mettenleiter TC (2006). Intriguing interplay between viral proteins during herpesvirus assembly or: the herpesvirus assembly puzzle.. Vet Microbiol.

[55] Mettenleiter TC, Klupp BG, Granzow H (2006). Herpesvirus assembly: a tale of two membranes.. Curr Opin Microbiol.

[56] Bortz E, Wang L, Jia Q, Wu T-T, Whitelegge JP (2007). Murine gammaherpesvirus 68 ORF52 encodes a tegument protein required for virion morphogenesis in the cytoplasm.. J Virol.

[57] White E (1993). Regulation of apoptosis by the transforming genes of the DNA tumor virus adenovirus.. Proc Soc Exp Biol Med.

[58] Roulston A, Marcellus RC, Branton PE (1999). Viruses and apoptosis.. Annu Rev Microbiol.

[59] Kaminskyy V, Zhivotovsky B (2010). To kill or be killed: how viruses interact with the cell death machinery.. J Intern Med.

[60] Yasukawa M, Inoue Y, Ohminami H, Terada K, Fujita S (1998). Apoptosis of CD4+ T lymphocytes in human herpesvirus-6 infection.. J Gen Virol.

[61] Roy DJ, Ebrahimi BC, Dutia BM, Nash AA, Stewart JP (2000). Murine gammaherpesvirus M11 gene product inhibits apoptosis and is expressed during virus persistence.. Arch Virol.

[62] Cheng EH, Nicholas J, Bellows DS, Hayward GS, Guo HG (1997). A Bcl-2 homolog encoded by Kaposi sarcoma-associated virus, human herpesvirus 8, inhibits apoptosis but does not heterodimerize with Bax or Bak.. Proc Natl Acad Sci U S A.

[63] Cleary ML, Smith SD, Sklar J (1986). Cloning and structural analysis of cDNAs for bcl-2 and a hybrid bcl-2/immunoglobulin transcript resulting from the t(14;18) translocation.. Cell.

[64] Ku B, Woo J-S, Liang C, Lee K-H, Hong H-S (2008). Structural and biochemical bases for the inhibition of autophagy and apoptosis by viral BCL-2 of murine gamma-herpesvirus 68.. PLoS Pathog.

[65] Sinha S, Colbert CL, Becker N, Wei Y, Levine B (2008). Molecular basis of the regulation of Beclin 1-dependent autophagy by the gamma-herpesvirus 68 Bcl-2 homolog M11.. Autophagy.

[66] Brown HJ, Song MJ, Deng H, Wu T-T, Cheng G (2003). NF-kappaB inhibits gammaherpesvirus lytic replication.. J Virol.

[67] Krug LT, Moser JM, Dickerson SM, Speck SH (2007). Inhibition of NF-kappaB activation in vivo impairs establishment of gammaherpesvirus latency.. PLoS Pathog.

[68] An J, Sun Y, Sun R, Rettig MB (2003). Kaposi's sarcoma-associated herpesvirus encoded vFLIP induces cellular IL-6 expression: the role of the NF-kappaB and JNK/AP1 pathways.. Oncogene.

[69] Karin M, Ben-Neriah Y (2000). Phosphorylation Meets Ubiquitination: The Control of NF-κB Activity.. Annu Rev Immunol.

[70] Sadagopan S, Sharma-Walia N, Veettil MV, Raghu H, Sivakumar R (2007). Kaposi's sarcoma-associated herpesvirus induces sustained NF-kappaB activation during de novo infection of primary human dermal microvascular endothelial cells that is essential for viral gene expression.. J Virol.

[71] Grossmann C, Ganem D (2008). Effects of NFκB activation on KSHV latency and lytic reactivation are complex and context-dependent.. Virology.

[72] Kaye KM, Devergne O, Harada JN, Izumi KM, Yalamanchili R (1996). Tumor necrosis factor receptor associated factor 2 is a mediator of NF-kappa B activation by latent infection membrane protein 1, the Epstein-Barr virus transforming protein.. Proc Natl Acad Sci U S A.

[73] Luftig M, Prinarakis E, Yasui T, Tsichritzis T, Cahir-McFarland E (2003). Epstein-Barr virus latent membrane protein 1 activation of NF-kappaB through IRAK1 and TRAF6.. Proc Natl Acad Sci U S A.

[74] Amici C, Rossi A, Costanzo A, Ciafrè S, Marinari B (2006). Herpes simplex virus disrupts NF-kappaB regulation by blocking its recruitment on the IkappaBalpha promoter and directing the factor on viral genes.. J Biol Chem.

[75] Hong GK, Kumar P, Wang L, Damania B, Gulley ML (2005). Epstein-Barr virus lytic infection is required for efficient production of the angiogenesis factor vascular endothelial growth factor in lymphoblastoid cell lines.. J Virol.

[76] Chandriani S, Ganem D (2007). Host transcript accumulation during lytic KSHV infection reveals several classes of host responses.. PLoS ONE.

[77] Chaudhury A, Chander P, Howe PH (2010). Heterogeneous nuclear ribonucleoproteins (hnRNPs) in cellular processes: Focus on hnRNP E1's multifunctional regulatory roles.. RNA.

[78] Collier B, Goobar-Larsson L, Sokolowski M, Schwartz S (1998). Translational inhibition in vitro of human papillomavirus type 16 L2 mRNA mediated through interaction with heterogenous ribonucleoprotein K and poly(rC)-binding proteins 1 and 2.. J Biol Chem.

[79] Nishimura K, Ueda K, Guwanan E, Sakakibara S, Do E (2004). A posttranscriptional regulator of Kaposi's sarcoma-associated herpesvirus interacts with RNA-binding protein PCBP1 and controls gene expression through the IRES.. Virology.

[80] Pacheco A, Reigadas S, Martínez-Salas E (2008). Riboproteomic analysis of polypeptides interacting with the internal ribosome-entry site element of foot-and-mouth disease viral RNA.. Proteomics.

[81] Perera R, Daijogo S, Walter BL, Nguyen JHC, Semler BL (2007). Cellular protein modification by poliovirus: the two faces of poly(rC)-binding protein.. J Virol.

[82] Walter BL, Parsley TB, Ehrenfeld E, Semler BL (2002). Distinct poly(rC) binding protein KH domain determinants for poliovirus translation initiation and viral RNA replication.. J Virol.

[83] Spångberg K, Schwartz S (1999). Poly(C)-binding protein interacts with the hepatitis C virus 5′ untranslated region.. J Gen Virol.

[84] Hartwell LH, Hopfield JJ, Leibler S, Murray AW (1999). From molecular to modular cell biology.. Nature.

[85] Barabási A-L, Oltvai ZN (2004). Network biology: understanding the cell's functional organization.. Nat Rev Genet.

[86] MacPherson JI, Dickerson JE, Pinney JW, Robertson DL (2010). Patterns of HIV-1 Protein Interaction Identify Perturbed Host-Cellular Subsystems.. PLoS Comput Biol.

[87] König R, Stertz S, Zhou Y, Inoue A, Hoffmann H-H (2010). Human host factors required for influenza virus replication.. Nature.

[88] Miller G, Fields BN, Knipe DM, Chanock RM, Hirsch MS, Melnick JL (1990). Epstein-Barr virus: biology, pathogenesis and medical aspects.. Fields Virology.

[89] Sullivan RJ, Pantanowitz L, Casper C, Stebbing J, Dezube BJ (2008). HIV/AIDS: epidemiology, pathophysiology, and treatment of Kaposi sarcoma-associated herpesvirus disease: Kaposi sarcoma, primary effusion lymphoma, and multicentric Castleman disease.. Clin Infect Dis.

[90] Guthrie C, Fink GR (1991). Guide to yeast genetics and molecular biology.. Meth Enzymol.

[91] Ding X, Zhang Y, Song W-Y (2007). Use of rolling-circle amplification for large-scale yeast two-hybrid analyses.. Methods Mol Biol.

[92] Soellick TR, Uhrig JF (2001). Development of an optimized interaction-mating protocol for large-scale yeast two-hybrid analyses.. Genome Biol.

[93] Ma H, Kunes S, Schatz PJ, Botstein D (1987). Plasmid construction by homologous recombination in yeast.. Gene.

[94] Krueger U, Bergauer T, Kaufmann B, Wolter I, Pilk S (2007). Insights into effective RNAi gained from large-scale siRNA validation screening.. Oligonucleotides.

[95] Matveeva O, Nechipurenko Y, Rossi L, Moore B, Saetrom P (2007). Comparison of approaches for rational siRNA design leading to a new efficient and transparent method.. Nucleic Acids Res.

[96] Huesken D, Lange J, Mickanin C, Weiler J, Asselbergs F (2005). Design of a genome-wide siRNA library using an artificial neural network.. Nat Biotechnol.

[97] Mukherji M, Bell R, Supekova L, Wang Y, Orth AP (2006). Genome-wide functional analysis of human cell-cycle regulators.. Proc Natl Acad Sci U S A.

[98] Kerrien S, Alam-Faruque Y, Aranda B, Bancarz I, Bridge A (2007). IntAct–open source resource for molecular interaction data.. Nucleic Acids Res.

[99] Chatr-aryamontri A, Ceol A, Palazzi LM, Nardelli G, Schneider MV (2007). MINT: the molecular INTeraction database.. Nucleic Acids Res.

[100] Orchard S, Kerrien S, Jones P, Ceol A, Chatr-Aryamontri A (2007). Submit your interaction data the IMEx way: a step by step guide to trouble-free deposition.. Proteomics.

[101] Maere S, Heymans K, Kuiper M (2005). BiNGO: a Cytoscape plugin to assess overrepresentation of gene ontology categories in biological networks.. Bioinformatics.

