# Supplementary material for: An Integrated Approach to Elucidate the Intra-Viral and Viral-Cellular Protein Interaction Networks of a Gamma-Herpesvirus
Source: PLoS Pathog. 2011 Oct 20;7(10):e1002297. doi: 10.1371/journal.ppat.1002297 (PMC3197595; doi:10.1371/journal.ppat.1002297)
Supplement: Table S1 — MHV-68 intra-viral protein-protein interactions. (PDF) [file ppat.1002297.s009.pdf]

Table S1. MHV-68 intra-viral protein-protein interactions

Bait	UniprotKB	RefSeq	Bait aa	Prey	UniprotKB	RefSeq	Prey aa	Hits	CO-IP	IP (B->P)	IP (P->B)	co-localization	Interaction detected between orthoogous proteins (PMID [virus])
ORF6a	O41928	IP_044846.	1-632	ORF4	O41927	NP_044845.1	1-388	3	+	-	+		
ORF33	O41949	IP_044870.	1-327	ORF37	O41954	NP_044875.1	1-486	4	-			-	
ORF33	O41949	IP_044870.	1-327	ORF38	O41955	NP_044876.1	1-75	4	+	+	-	+	16014918,14557627,19730696 [HSV-1] 19730696 [mCMV] 19730696 [EBV], 0962080 [HCMV], 20205919 [VZV]
ORF33	O41949	IP_044870.	1-327	ORF44	P88981	NP_044881.1	1-776	4	-			+	
ORF33	O41949	IP_044870.	1-327	ORF45	P88983	NP_044882.1	1-206	4	+	+	+	+	18321973 [KSHV]
ORF34	O41951	IP_044872.	1-332	ORF52	P88989	NP_044889.1	1-135	4	+	+	+		16339411 [KSHV]
ORF38	O41955	IP_044876.	1-75	ORF33	O41949	NP_044870.1	1-327	4	+	-	+	+	16014918,14557627,19730696 [HSV-1] 19730696 [mCMV] 19730696 [EBV], 20205919 [VZV]
ORF40	O41957	IP_044878.	1-610	ORF68	O41969		1-460	4	+	-	+	+	
ORF45	P88983	IP_044882.	1-206	ORF32	O41948	NP_044869.1	1-444	4	-			+	
ORF45	P88983	IP_044882.	1-206	ORF33	O41949	NP_044870.1	1-327	4	+	+	+	+	18321973 [KSHV]
ORF45	P88983	IP_044882.	1-206	ORF52	P88989	NP_044889.1	1-135	4	+	+	-	+	18321973 [KSHV]
ORF45	P88983	IP_044882.	1-206	ORF67	O41968	NP_044905.1	1-226	4	-			-	
ORF45	P88983	IP_044882.	1-206	ORF68	O41969		1-460	4	+	+	-	+	
ORF56	P88992	IP_044893.	1-835	ORF39	O41956	NP_044877.1	1-383	4	+	+	+	+	
ORF56	P88992	IP_044893.	1-835	ORF40	O41957	NP_044878.1	1-610	4	low			+	10580049 [EBV], 10501495,7931156,9344911 [HSV-1]
ORF56	P88992	IP_044893.	1-835	ORF44	P88981	NP_044881.1	1-776	4	low			-	10580049 [EBV], 10075707,10501495 [HSV-1]
ORF58	P88995	IP_044896.	1-347	ORF26	P88980	NP_044864.1	1-299	4	low			+	
ORF58	P88995	IP_044896.	1-347	ORF27	O41944	NP_044865.1	1-254	4	+	+	-	+	19730696 [EBV], 16339411 [KSHV]
ORF62	O41963		1-380	ORF59	P88996	NP_044897.1	1-394	4	+	+	+	+	
ORF62	O41963		1-380	ORF60	P88997	NP_044898.1	1-305	4	+	+	+	+	
ORF64a	O41965	O41963	1-314	ORF63	O41964		1-938	2	low			-	16014918,18602131 [HSV-1], 18321973 [KSHV],19730696 [EBV], 16339411 [VZV]
ORF68	O41969		1-460	ORF67	O41968	NP_044905.1	1-226	4	+	-	+	+	20205919 [VZV]
ORF68	O41969		1-460	ORF68	O41969		1-460	4	-			+	20205919, 16339411, 19730696 [EBV]
ORF75b1	O41977	IP_044916.	1-750	ORF75b2	O41977	NP_044916.1	751-1275	4	-	+	-	+	
ORF67	O41968	IP_044905.	1-226	ORF69	O41970	NP_044907.1	1-292	4	+	+	+	+	10627546,11507225,15731273,19730696 [HSV-1], 15003866,15731265,17446270, 19730696 [EBV], 17005637, 19730696 [mCMV], 17872514,19153235 [HCMV], 20205919, 16339411 [VZV]

Note1: a, b1,b2: ORF fragment clones

Note2: low: low expression level for one of the partner
