# Supplementary material for: An Integrated Approach to Elucidate the Intra-Viral and Viral-Cellular Protein Interaction Networks of a Gamma-Herpesvirus
Source: PLoS Pathog. 2011 Oct 20;7(10):e1002297. doi: 10.1371/journal.ppat.1002297 (PMC3197595; doi:10.1371/journal.ppat.1002297)
Supplement: Table S2 — MHV-68 virus-cellular protein-protein interactions. (PDF) [file ppat.1002297.s010.pdf]

Table S2. MHV-68 viral-cellular protein-protein interactions

Bait (Vira)	Prey (Cellular)										
Name	Gene Symbol	Gene ID	RefSeq	UniProt	start	end	insert size	Retest result	rank	scr(N)	p-val
M11	ALB	213	NP_000468.1	P02768-1	1506	1808	302	Positive	76	3	7.13E-01
M11	BECN1	8678	NP_003757.1	Q14457	259	833	574	Positive	13	41	6.19E-02
M11	C12orf35	55196	NP_060639.3	Q9HCM1	1185	1653	468	Positive			
M11	COPS5	10987	NP_006828.2	Q92905	480	1119	639	Weak Positive	11	43	5.10E-02
M11	DST	667			3711	4202	491	Positive	62	9	4.84E-01
M11	FTL	2512	NP_000137.2	P02792	309	723	414	Positive			
M11	IQGAP2	10788	NP_006624.2	Q13576-1	939	1443	504	Positive	68	6	5.79E-01
M11	KDM5A	5927	NP_001036068.1	P29375-1	4221	4843	622	Positive	65	7	5.45E-01
M11	NUP35	129401	NP_612142.2	Q8NFH5	82	543	461	Positive			
M11	PAH	5053	NP_000268.1	P00439	436	742	306	Positive			
M11	PAIP1	10605	NP_899152.1		1186	1565	379	Positive	44	17	3.03E-01
M11	SPTAN1	6709	NP_003118.2	Q13813-1	4943	5377	434	Positive	49	15	3.43E-01
M11	UTRN	7402	NP_009055.2	P46939	5925	6740	815	Positive	61	9	4.84E-01
M3	GRINA	2907	NP_001009184.1	Q7Z429	703	1247	544	Weak Positive			
M3	UBC	7316	NP_066289.2	P62988	1728	2132	404	Weak Positive	67	7	5.45E-01
M9	ACTN1	87	NP_001093.1	P12814	1203	1971	768	Positive	16	39	7.08E-02
M9	GOLGA2	2801	NP_004477.3	Q08379-1	1198	1906	708	Positive	74	4	6.67E-01
M9	HSP90B1	7184	NP_003290.1	P14625	1542	2371	829	Positive	46	17	3.03E-01
ORF4	HRG	3273	NP_000403.1	P04196	806	1486	680	Positive			
ORF10	ACBD3	64746	NP_073572.2	Q9H3P7	539	1019	480	Positive	89	0	1.00E+00
ORF10	ALB	213	NP_000468.1	P02768-1	106	510	404	Positive	76	3	7.13E-01
ORF10	ERBB3	2065		P21860	1362	1696	334	Positive	35	22	2.28E-01
ORF10	ETAA1	54465	NP_061875.2	Q9NY74	547	1198	651	Positive			
ORF10	FGB	2244	NP_005132.2	P02675	868	1392	524	Positive	80	2	7.98E-01
ORF10	FN1	2335	NP_002017.1	P02751-3	874	1361	487	Positive	33	24	2.01E-01
ORF10	GRIPAP1	56850	NP_064522.3	Q4V328-1	31	605	574	Positive			
ORF11	CCDC144B	284047	NP_872374.3	Q3MJ40-1	925	1609	684	Weak Positive			
ORF11	DIAPH2	1730	NP_009293.1	O60879-2	1775	2235	460	Positive			
ORF11	NHSL1	57224	NP_001137532.1		3093	3431	338	Positive			
ORF11	PHACTR4	65979	NP_076412.3	Q8IZ21-2	1896	2270	374	Positive			
ORF11	SYNPO2	171024	NP_001122406.1	B2RWP6	1102	1625	523	Positive			
ORF11	WASF1	8936	NP_001020107.1	Q92558	1164	1900	736	Positive	10	50	2.53E-02
ORF11	WASL	8976	NP_003932.3	O00401	1057	1303	246	Positive	20	34	1.04E-01
ORF20	CEP110	11064	NP_008949.4	Q7Z7A1	5622	6081	459	Positive			
ORF23	C10orf118	55088	NP_060487.2	Q7Z3E2	1150	1880	730	Positive			
ORF23	CCDC99	54908	NP_060255.3	Q96EA4-1	732	1166	434	Positive			

ORF23	CDK5RAP2	55755	NP_060719.4	Q96SN8-1	328	1091	763	Positive	94	0	1.00E+00
ORF23	CNTLN	54875	NP_060208.2	Q9NXG0-2	1592	2059	467	Positive			
ORF23	MYH10	4628	NP_005955.1	P35580-1	4584	4921	337	Positive	69	6	5.79E-01
ORF23	PPFIBP2	8495	NP_003612.2	Q8ND30	626	1038	412	Positive			
ORF26	ANKRD17	26057	NP_115593.3	O75179	5499	6204	705	Positive			
ORF26	ARHGEF12	23365	NP_056128.1	Q9NZN5-1	3739	4160	421	Positive	71	6	5.79E-01
ORF26	BAT3	7917	NP_004630.3	P46379-1	2573	3402	829	Positive	63	9	4.84E-01
ORF26	CALCOCO1	57658	NP_065949.1	Q9P1Z2-1	1016	1554	538	Positive			
ORF26	CARD11	84433	NP_115791.3	Q9BXL7	3829	4250	421	Positive	30	25	1.88E-01
ORF26	CTNNB1	1499	NP_001895.1	P35222-1	1361	2145	784	Positive	4	70	7.47E-04
ORF26	DSCR3	10311	NP_006043.1	O14972	226	728	502	Positive			
ORF26	EMILIN1	11117	NP_008977.1	Q9Y6C2	1898	2531	633	Positive	39	19	2.71E-01
ORF26	GOLGB1	2804	NP_004478.3	Q14789	5492	6192	700	Positive	101	0	1.00E+00
ORF26	HSP90AA1	3320	NP_001017963.2	P07900-2	1549	2267	718	Positive	28	25	1.88E-01
ORF26	LOC100127894	100127894	XP_001723926.1		1224	1880	656	Positive			
ORF26	PSMB5	5693	NP_002788.1	P28074	35	473	438	Positive	78	3	7.13E-01
ORF26	TRIM37	4591	NP_056109.1	O94972-1	1940	2400	460	Positive	95	0	1.00E+00
ORF28	FGA	2243	NP_000499.1	P02671-1	436	906	470	Positive	84	1	8.51E-01
ORF28	STX17	55014	NP_060389.2	P56962	448	932	484	Positive			
ORF30	COPS2	9318	NP_004227.1	P61201-1	29	760	731	Positive	70	6	5.79E-01
ORF30	COPS5	10987	NP_006828.2	Q92905	480	1119	639	Positive	11	43	5.10E-02
ORF30	FGA	2243	NP_068657.1	P02671-2	106	563	457	Positive	84	1	8.51E-01
ORF30	GRIPAP1	56850	NP_064522.3	Q4V328-1	35	836	801	Positive			
ORF30	KPNA4	3840	NP_002259.1	O00629	633	1328	695	Positive	97	0	1.00E+00
ORF30	PDE4DIP	9659	NP_001002811.1	Q5VU43-2	2075	2438	363	Weak Positive			
ORF30	PSMD7	5713	NP_002802.2	P51665	555	1090	535	Positive	51	15	3.43E-01
ORF30	SDC2	6383	NP_002989.2	P34741	771	1196	425	Positive	53	14	3.62E-01
ORF30	SHROOM1	134549	NP_597713.2	Q2M3G4-2	1550	2396	846	Positive			
ORF30	SPTBN1	6711	NP_842565.2	Q01082-3	1543	1936	393	Positive	18	36	8.93E-02
ORF30	STX8	9482	NP_004844.1	Q9UNK0	180	746	566	Positive	93	0	1.00E+00
ORF31	BMPER	168667	NP_597725.1	Q8N8U9	950	1355	405	Positive			
ORF31	CALCOCO2	10241	NP_005822.1	Q13137	1030	1375	345	Positive	87	1	8.51E-01
ORF31	HRG	3273	NP_000403.1	P04196	853	1482	629	Weak Positive			
ORF31	KDM4B	23030	NP_055830.1	O94953	1979	2391	412	Positive			
ORF31	KCMF1	56888	NP_064507.3	Q9P0J7	560	1372	812	Positive			
ORF31	PCSK6	5046	NP_002561.1	P29122-1	2210	2747	537	Positive			
ORF31	POGZ	23126	NP_997054.1	Q7Z3K3-2	1317	1929	612	Positive	42	18	2.86E-01
ORF31	RAP80	51720	NP_057374.3	Q96RL1-1	1139	1700	561	Positive	21	34	1.04E-01
ORF31	REST	5978	NP_005603.2	Q13127-1	1251	1758	507	Positive	25	31	1.29E-01

ORF31	TAX1BP1	8887	NP_006015.4	Q86VP1-1	1757	2354	597	Positive	47	17	3.03E-01
ORF31	ZFR	51663	NP_057191.2	Q96KR1	1570	2119	549	Positive			
ORF31	ZNF638	27332	NP_055312.2	Q14966-1	5431	6231	800	Positive			
ORF33	ACADM	34	NP_000007.1	P11310-1	272	772	500	Positive			
ORF33	C14orf104	55172	NP_060609.2	Q9NVR5-1	1277	2123	846	Positive			
ORF33	CD97	976	NP_001775.2	P48960-2	269	480	211	Positive			
ORF33	CRIM1	51232	NP_057525.1	Q9NZV1	2103	2776	673	Positive			
ORF33	F9	2158	NP_000124.1	P00740	82	704	622	Positive			
ORF33	FN1	2335	NP_997639.1	P02751-10	5372	5686	314	Positive	33	24	2.01E-01
ORF33	GKAP1	80318	NP_079487.2	Q5VSY0-2	695	998	303	Positive			
ORF33	HNF4A	3172	NP_849180.1	P41235-2	8	411	403	Positive	56	12	4.05E-01
ORF33	ITGB3	3690	NP_000203.2	P05106-1	1523	1968	445	Positive	29	26	1.76E-01
ORF33	LAMA2	3908	NP_000417.2	P24043	2196	2869	673	Positive			
ORF33	MATN2	4147	NP_002371.3	O00339-1	1323	1984	661	Positive			
ORF33	NOTCH2NL	388677	NP_982283.2	Q7Z3S9-1	603	1062	459	Positive			
ORF33	NR1H4	9971	NP_005114.1	Q96RI1-2	357	776	419	Positive	66	7	5.45E-01
ORF33	THRSP	7069	NP_003242.1	Q92748	10	449	439	Positive			
ORF33	ZNF507	22847	NP_055725.2	Q8TCN5-1	347	434	87	Positive			
ORF33	ZNF655	79027	NP_001009960.1	Q8N720-1	862	1064	202	Positive	75	4	6.67E-01
ORF34	CREBBP	1387	NP_004371.2	Q92793	2357	2763	406	Positive	3	72	4.48E-04
ORF34	LOC728047	728047	XP_001126912.1		185	962	777	Positive			
ORF34	PCBP1	5093	NP_006187.2	Q15365	417	858	441	Positive	26	30	1.38E-01
ORF34	PCBP2	5094	NP_005007.2	Q6IPF4	571	1225	654	Positive	36	22	2.28E-01
ORF34	WNK1	65125	NP_061852.1	Q9H4A3-1	6987	7513	526	Positive	81	2	7.98E-01
ORF35	C12orf35	55196	NP_060639.3	Q9HCM1	1107	1684	577	Weak Positive			
ORF35	DST	667	NP_056363.2	Q03001-8	3711	4202	491	Positive	62	9	4.84E-01
ORF35	KRT8	3856	NP_002264.1	P05787	509	1207	698	Positive	22	33	1.12E-01
ORF35	LUC7L2	51631	NP_057103.2	Q9Y383-1	560	1141	581	Positive			
ORF35	MT2A	4502	NP_005944.1	P02795	38	314	276	Positive	86	1	8.51E-01
ORF35	STX8	9482	NP_004844.1	Q9UNK0	180	746	566	Positive	93	0	1.00E+00
ORF37	ADNP	23394	NP_056154.1	Q9H2P0	1440	2123	683	Positive			
ORF37	ANKRD17	26057	NP_115593.3	O75179-1	714	1546	832	Positive			
ORF37	RBAK	57786	NP_066986.1	Q9NYW8	1983	2033	50	Positive			
ORF37	RSN	6249	NP_002947.1	P30622-1	3529	3791	262	Positive	82	2	7.98E-01
ORF38	SDCBP	6386	NP_001007068.1	O00560-1	232	709	477	Positive	50	15	3.43E-01
ORF38	UBE2I	7329	NP_003336.1	P63279	52	603	551	Weak Positive	2	75	1.49E-04
ORF39	TRAM1	23471	NP_055109.1	Q15629	70	539	469	Positive	55	12	4.05E-01
ORF40	ACTN4	81	NP_004915.2	O43707	1412	2227	815	Positive	24	32	1.20E-01
ORF40	ANKRD50	57182	NP_065070.1	Q9ULJ7	1903	2693	790	Positive			

ORF40	CGN	57530	NP_065821.1		1798	2433	635	Positive	38	20	2.54E-01
ORF40	DVL1	1855	NP_004412.2	O14640-2	655	1417	762	Positive			
ORF40	CCDC158	339965	NP_001036249.1	Q5M9N0-1	1185	1675	490	Positive			
ORF40	FUBP1	8880	NP_003893.2	Q96AE4-1	173	763	590	Positive			
ORF40	GGA2	23062	NP_055859.1	Q9UJY4	514	1258	744	Positive	58	12	4.05E-01
ORF40	GOLGA2	2801	NP_004477.3	Q08379-1	877	1680	803	Positive	74	4	6.67E-01
ORF40	GOLGB1	2804	NP_004478.3	Q14789	7208	7990	782	Positive	101	0	1.00E+00
ORF40	HSP90B1	7184	NP_003290.1	P14625	1014	1822	808	Positive	46	17	3.03E-01
ORF40	HSPA4	3308	NP_002145.3	P34932	2024	2565	541	Positive	19	36	8.93E-02
ORF40	HSPA5	3309	NP_005338.1	P11021	1242	1769	527	Positive	7	59	8.32E-03
ORF40	KTN1	3895	NP_891556.1	Q86UP2-1	1187	1995	808	Positive			
ORF40	DVL1	1855	NP_004412.2	O14640-2	1180	1788	608	Positive			
ORF40	MYH11	4629	NP_002465.1	P35749	3137	3767	630	Positive			
ORF40	MYH14	79784	NP_079005.3	Q7Z406-1	4097	4820	723	Positive			
ORF40	RBAK	57786	NP_066986.1	Q9NYW8	1868	2329	461	Positive			
ORF40	RBM5	10181	NP_005769.1	P52756-1	1288	2161	873	Positive			
ORF40	TRIP11	9321	NP_004230.2	Q15643	1769	2599	830	Positive			
ORF40	ZRANB1	54764	NP_060050.2	Q9UGI0	829	1427	598	Positive	41	19	2.71E-01
ORF42	DLG5	9231	NP_004738.3	Q8TDM6-1	4125	4550	425	Positive	43	18	2.86E-01
ORF42	FTL	2512	NP_000137.2	P02792	523	560	37	Positive			
ORF42	MPHOSPH1	9585	NP_057279.2	Q96Q89-3	4043	4765	722	Positive			
ORF42	NFE2L2	4780	NP_006155.2	Q16236-1	7	512	505	Positive	100	0	1.00E+00
ORF42	NKRF	55922	NP_060014.2	O15226	382	1107	725	Positive			
ORF42	PPP2R5A	5525	NP_006234.1	Q15172	976	1627	651	Positive	12	44	4.62E-02
ORF42	SORBS2	8470	NP_003594.3	O94875-2	849	1404	555	Positive	59	11	4.33E-01
ORF44	RPA1	6117	NP_002936.1	P27694	1	449	448	Positive	60	10	4.59E-01
ORF45	CCDC123	84902	NP_116205.3	Q96ST8-1	1667	2194	527	Positive			
ORF45	F5	2153	NP_000121.2	P12259	2872	3549	677	Positive			
ORF45	FLJ25778	254048	NP_775840.3	Q6ZU65-1	302	1158	856	Positive			
ORF45	HNRNPC	3183	NP_004491.2	P07910-2	517	942	425	Positive	27	28	1.54E-01
ORF45	PRPF3	9129	NP_004689.1	O43395-1	1143	1696	553	Positive	91	0	1.00E+00
ORF45	UTP14A	10813	NP_006640.2	Q9BVJ6-1	9	739	730	Positive			
ORF45	ZFR	51663	NP_057191.2	Q96KR1	563	1190	627	Positive			
ORF48	C4B	721	NP_001002029.3	P0C0L5	663	1368	705	Positive			
ORF48	DST	667	NP_056363.2	Q03001	14372	15208	836	Positive	62	9	4.84E-01
ORF48	GOLGB1	2804	NP_004478.3	Q14789	3879	4612	733	Positive	101	0	1.00E+00
ORF48	LUZP1	7798	NP_361013.3	Q86V48-1	407	749	342	Positive			
ORF49	APOB	338	NP_000375.2	P04114	6821	7247	426	Positive			
ORF49	BAT2L	84726	NP_037450.2		562	946	384	Positive	72	5	6.19E-01

ORF49	DDX23	9416	NP_004809.2	Q9BUQ8	2580	2897	317	Positive	77	3	7.13E-01
ORF49	EHMT2	10919	NP_079532.5	Q96KQ7-2	1981	2261	280	Positive	83	2	7.98E-01
ORF49	LAMB2	3913	NP_002283.3	P55268	4043	4705	662	Positive			
ORF49	LOC399491	339047	NP_848636.1	Q86VD5	837	1292	455	Positive			
ORF49	PCF11	51585	NP_056969.2	O94913	398	457	59	Positive			
ORF49	PPFIBP1	8496	NP_003613.2	Q86W92-2	1222	1342	120	Positive			
ORF49	SUPT6H	6830	NP_003161.2	Q7KZ85-1	4805	5204	399	Positive	54	14	3.62E-01
ORF49	TIAL1	7073	NP_003243.1	Q01085	846	1543	697	Positive	64	7	5.45E-01
ORF49	ZNF44	51710	NP_057348.3		1422	1877	455	Positive			
ORF50	APOA1	335	NP_000030.1	P02647	455	835	380	Positive	99	0	1.00E+00
ORF50	APOB	338	NP_000375.2	P04114	6980	7512	532	Positive			
ORF50	APOOL	139322	NP_940852.3	Q6UXV4	17	568	551	Positive			
ORF50	COPB2	9276	NP_004757.1	P35606	2028	2383	355	Positive	48	16	3.22E-01
ORF50	FLNA	2316	NP_001447.2	P21333-2	5939	6588	649	Positive	9	52	1.99E-02
ORF50	WNK1	65125	NP_061852.2	Q9H4A3-1	4095	4459	364	Positive	81	2	7.98E-01
ORF52	LOC100127894	100127894	XP_001723926.1		1525	1891	366	Positive			
ORF53	DDX24	57062	NP_065147.1	Q9GZR7	20	746	726	Positive			
ORF53	STX17	55014	NP_060389.2	P56962	448	932	484	Positive			
ORF54	BAT2D1	23215	NP_055987.2		7703	8127	424	Positive			
ORF54	HECTD1	25831	NP_056197.2	Q9ULT8	2771	3161	390	Positive	85	1	8.51E-01
ORF54	POGZ	23126	NP_055915.2	Q7Z3K3-1	329	888	559	Positive	42	18	2.86E-01
ORF54	PUF60	22827	NP_510965.1	Q9UHX1-1	766	1376	610	Positive	96	0	1.00E+00
ORF54	SSFA2	6744	NP_006742.2	P28290-3	262	700	438	Positive			
ORF54	UBAP1	51271	NP_057609.2	Q9NZ09	859	1648	789	Positive			
ORF54	WNK1	65125	NP_061852.3	Q9H4A3-1	4095	4459	364	Positive	81	2	7.98E-01
ORF55	ALB	213	NP_000468.1	P02768-1	1645	2051	406	Positive	76	3	7.13E-01
ORF55	CEP63	80254	NP_079456.2	Q96MT8-1	939	1480	541	Positive			
ORF55	DST	667			11721	12344	623	Positive	62	9	4.84E-01
ORF55	FGB	2244	NP_005132.2	P02675	115	641	526	Weak Positive	80	2	7.98E-01
ORF55	INTS6	26512	NP_001035026.1	Q9UL03	2490	2915	425	Positive	15	40	6.63E-02
ORF55	IPO7	10527	NP_006382.1	O95373	1985	2544	559	Positive	8	54	1.61E-02
ORF55	LARP2	55132	NP_835144.1	Q659C4	831	1412	581	Positive			
ORF55	NASP	4678	NP_002473.2	P49321-1	650	961	311	Positive	57	12	4.05E-01
ORF55	SMC6	79677	NP_078900.1	Q96SB8-1	771	1140	369	Positive			
ORF55	SPTBN1	6711	NP_842565.2	Q01082-3	5458	5877	419	Positive	18	36	8.93E-02
ORF55	TRIP11	9321	NP_004230.2	Q15643	3869	4524	655	Positive			
ORF55	UBAC1	10422	NP_057256.2	Q9BSL1	545	926	381	Positive			
ORF55	YWHAE	7531	NP_006752.1	P62258	416	875	459	Positive	1	81	
ORF56	MCC	4163	NP_002378.1	P23508-1	1154	1464	310	Positive	98	0	1.00E+00

ORF56	NBPF11	200030	NP_899228.3	B1AKG1	769	1364	595	Positive			
ORF56	PDE4DIP	9659	NP_001002811.1	Q5VU43-2	1458	2150	692	Positive			
ORF56	SPTBN2	6712	NP_008877.1	O15020	2850	3697	847	Positive			
ORF56	USPL1	10208	NP_005791.3	Q5W0Q7-1	2556	3298	742	Positive			
ORF58	ATF6	22926	NP_031374.2	P18850	853	1590	737	Positive	92	0	1.00E+00
ORF58	DDX24	57062	NP_065147.1	Q9GZR7	20	746	726	Positive			
ORF58	IFITM1	8519	NP_003632.3	P13164	72	510	438	Positive			
ORF58	LRP5	4041	NP_002326.2	O75197	3861	4478	617	Weak Positive	90	0	1.00E+00
ORF58	LTBR	4055	NP_002333.1	P36941	435	929	494	Weak Positive	79	3	7.13E-01
ORF58	MFSD2	84879	NP_116182.2	Q8NA29-2	733	1120	387	Positive			
ORF58	PCDH12	51294	NP_057664.1	Q9NPG4	2869	3590	721	Positive			
ORF58	SERINC5	256987	NP_840060.1	Q86VE9-1	1123	1369	246	Positive			
ORF58	SLC43A1	8501	NP_003618.1	O75387-1	913	1475	562	Positive			
ORF58	SPCS2	9789	NP_055567.2	Q15005	21	510	489	Weak Positive			
ORF58	SREBF1	6720	NP_001005291.1	P36956-4	1517	2018	501	Weak Positive	40	19	2.71E-01
ORF58	TEX2	55852	NP_060939.3	Q8IWB9-2	732	1502	770	Positive			
ORF58	WNK1	65125	NP_061852.3	Q9H4A3-1	4301	4459	158	Positive	81	2	7.98E-01
ORF59	ANKHD1	54882	NP_078944.2	Q8IWZ3-2	1084	1799	715	Positive			
ORF59	DCTN1	1639	NP_004073.2	Q14203-1	2124	2794	670	Positive	14	41	6.19E-02
ORF59	KIAA0409	23378	NP_056139.1	O43159	10	422	412	Positive			
ORF59	NUMA1	4926	NP_006176.2	Q14980-1	1543	2121	578	Positive	31	25	1.88E-01
ORF59	PARP1	142	NP_001609.2	P09874	1551	2127	576	Positive	6	60	6.93E-03
ORF59	ZZZ3	26009	NP_056349.1	Q8IYH5-1	1316	2055	739	Positive			
ORF60	PEG3	5178	NP_006201.1	Q9GZU2-1	1324	1961	637	Positive			
ORF61	FLNA	2316	NP_001447.2	P21333-2	1277	1422	145	Weak Positive	9	52	1.99E-02
ORF62	APOA1	335	NP_000030.1	P02647	383	835	452	Weak Positive	99	0	1.00E+00
ORF63	DCTN1	1639	NP_004073.2	Q14203-1	2124	2911	787	Positive	14	41	6.19E-02
ORF63	EIF3A	8661	NP_003741.1	Q14152	2075	2720	645	Positive	34	24	2.01E-01
ORF63	KRCC1	51315	NP_057702.1	Q9NPI7	421	1111	690	Positive			
ORF63	KTN1	3895	NP_891556.1	Q86UP2-1	2686	3418	732	Positive			
ORF63	MYSM1	114803			1009	1402	393	Positive			
ORF63	RPL5	6125	NP_000960.2	P46777	440	855	415	Positive	37	21	2.41E-01
ORF63	WNK1	65125	NP_061852.3	Q9H4A3-1	4176	4459	283	Positive	81	2	7.98E-01
ORF68	CASP8AP2	9994	NP_036247.1	Q9UKL3	5308	5794	486	Positive	23	33	1.12E-01
ORF68	PIAS1	8554	NP_057250.1	O75925	1236	1755	519	Positive	5	65	2.74E-03
ORF68	RAD54L2	23132	NP_055921.2	Q9Y4B4	1	558	557	Positive			
ORF68	TNIP1	10318	NP_006049.3	Q15025-1	1336	1650	314	Positive	32	25	1.88E-01
ORF68	TPP2	7174	NP_003282.2	P29144	3006	3657	651	Positive			
ORF68	UBE2I	7329	NP_003336.1	P63279	52	603	551	Positive	2	75	1.49E-04

ORF69	AKAP9	10142	NP_005742.4	Q99996-2	9434	9862	428	Positive	17	37	8.25E-02
ORF69	ALB	213	NP_000468.1	P02768-1	54	541	487	Positive	76	3	7.13E-01
ORF69	CDK5RAP2	55755	NP_060719.4	Q96SN8-1	346	1040	694	Positive	94	0	1.00E+00
ORF69	CNTLN	54875	NP_060208.2	Q9NXG0-2	298	888	590	Positive			
ORF69	DST	667	NP_056363.2	Q03001-8	13302	13975	673	Positive	62	9	4.84E-01
ORF69	GOLGB1	2804	NP_004478.3	Q14789	6813	7461	648	Positive	101	0	1.00E+00
ORF69	IPO5	3843	NP_002262.3	O00410-3	2022	2828	806	Positive	73	5	6.19E-01
ORF69	KIAA0376	23384	NP_056145.2	Q69YQ0	1661	2465	804	Positive	52	14	3.62E-01
ORF69	LMNA	4000	NP_733822.1	P02545-3	507	1247	740	Positive	45	17	3.03E-01
ORF69	MACF1	23499	NP_149033.2	Q96PK2-1	14407	14625	218	Positive	88	1	8.51E-01
ORF69	SPTBN1	6711	NP_003119.2	Q01082-1	4936	5730	794	Positive	18	36	8.93E-02
ORF69	WNK1	65125	NP_061852.3	Q9H4A3-1	3592	3952	360	Positive	81	2	7.98E-01
