# Supplementary material for: An Integrated Approach to Elucidate the Intra-Viral and Viral-Cellular Protein Interaction Networks of a Gamma-Herpesvirus
Source: PLoS Pathog. 2011 Oct 20;7(10):e1002297. doi: 10.1371/journal.ppat.1002297 (PMC3197595; doi:10.1371/journal.ppat.1002297)
Supplement: Table S3 — Priority rank, priority score, protein degree, and effect on MHV-68 replication of cellular proteins from the MHV-68-cellular protein interaction network. The effects of silencing expression of cellular proteins (Fig. S6C) on MHV-68 replication were annotated as following: enhanced MHV-68 replication (+), inhibited MHV-68 replication (−), cellular toxicity (toxic). (PDF) [file ppat.1002297.s011.pdf]

Table S3. Priority rank, protein degree, and priority score of cellular protein from the MHV-68-cellular PPI network.

Rank	Gene ID	Gene Name	Degree	Score	p-val	siRNA
1	7531	YWHAE	33	81	1.40E-05	Toxic
2	7329	UBE2I	26	75	1.69E-04	
3	1387	CREBBP	27	72	4.25E-04	
4	1499	CTNNB1	37	70	7.35E-04	+
5	8554	PIAS1	7	65	2.51E-03	+
6	142	PARP1	17	60	6.59E-03	
7	3309	HSPA5	14	59	7.78E-03	
8	10527	IPO7	2	54	1.57E-02	
9	2316	FLNA	23	52	1.99E-02	
10	8936	WASF1	4	50	2.49E-02	
11	5525	PPP2R5A	11	44	4.48E-02	
12	10987	COPS5	14	43	4.91E-02	+
13	8678	BECN1	5	41	5.87E-02	
14	1639	DCTN1	5	41	5.87E-02	
15	26512	INTS6	7	40	6.38E-02	
16	87	ACTN1	4	39	6.92E-02	
17	10142	AKAP9	5	37	8.11E-02	
18	6711	SPTBN1	3	36	8.79E-02	
19	3308	HSPA4	4	36	8.79E-02	
20	8976	WASL	11	34	1.02E-01	
21	51720	RAP80	6	34	1.02E-01	
22	3856	KRT8	6	33	1.10E-01	
23	9994	CASP8AP2	2	33	1.10E-01	-
24	81	ACTN4	3	32	1.18E-01	
25	5978	REST	3	31	1.27E-01	
26	5093	PCBP1	3	30	1.36E-01	+
27	3183	HNRNPC	3	28	1.55E-01	+
28	3320	HSP90AA1	32	25	1.88E-01	
29	3690	ITGB3	10	26	1.77E-01	Toxic
30	84433	CARD11	5	25	1.88E-01	-
31	4926	NUMA1	3	25	1.88E-01	
32	10318	TNIP1	2	25	1.88E-01	
33	2335	FN1	7	24	2.00E-01	
34	8661	EIF3A	5	24	2.00E-01	
35	2065	ERBB3	10	22	2.26E-01	
36	5094	PCBP2	3	22	2.26E-01	
37	6125	RPL5	3	21	2.41E-01	-
38	57530	CGN	1	20	2.55E-01	
39	11117	EMILIN1	1	19	2.71E-01	
40	6720	SREBF1	6	19	2.71E-01	
41	54764	ZRANB1	2	19	2.71E-01	
42	23126	POGZ	2	18	2.87E-01	
43	9231	DLG5	1	18	2.87E-01	
44	10605	PAIP1	1	17	3.04E-01	
45	4000	LMNA	3	17	3.04E-01	
46	8887	TAX1BP1	5	17	3.04E-01	-
47	7184	HSP90B1	4	17	3.04E-01	
48	9276	COPB2	1	16	3.22E-01	
49	6709	SPTAN1	10	15	3.41E-01	
50	6386	SDCBP	2	15	3.41E-01	
51	5713	PSMD7	3	15	3.41E-01	

52	23384	KIAA0376	1	14	3.61E-01	
53	6383	SDC2	5	14	3.61E-01	
54	6830	SUPT6H	1	14	3.61E-01	Toxic
55	23471	TRAM1	1	12	4.06E-01	
56	4678	NASP	2	12	4.06E-01	
57	3172	HNF4A	2	12	4.06E-01	
58	23062	GGA2	7	12	4.06E-01	
59	8470	SORBS2	5	11	4.30E-01	
60	6117	RPA1	18	10	4.56E-01	
61	7402	UTRN	6	9	4.83E-01	
62	667	DST	3	9	4.83E-01	
63	7917	BAT3	1	9	4.83E-01	
64	7073	TIAL1	1	7	5.44E-01	
65	5927	KDM5A	1	7	5.44E-01	
66	9971	NR1H4	3	7	5.44E-01	
67	7316	UBC	2	7	5.44E-01	
68	10788	IQGAP2	1	6	5.79E-01	
69	4628	MYH10	1	6	5.79E-01	-
70	9318	COPS2	2	6	5.79E-01	
71	23365	ARHGEF12	3	6	5.79E-01	
72	84726	BAT2L	1	5	6.17E-01	
73	3843	IPO5	1	5	6.17E-01	
74	2801	GOLGA2	8	4	6.65E-01	
75	79027	ZNF655	2	4	6.65E-01	
76	213	ALB	25	3	7.13E-01	
77	9416	DDX23	2	3	7.13E-01	+
78	5693	PSMB5	9	3	7.13E-01	
79	4055	LTBR	3	3	7.13E-01	
80	2244	FGB	2	2	7.98E-01	
81	65125	WNK1	2	2	7.98E-01	Toxic
82	6249	RSN	3	2	7.98E-01	
83	10919	EHMT2	4	2	7.98E-01	
84	2243	FGA	2	1	8.51E-01	
85	25831	HECTD1	1	1	8.51E-01	
86	4502	MT2A	4	1	8.51E-01	
87	10241	CALCOCO2	1	1	8.51E-01	
88	23499	MACF1	7	1	8.51E-01	
89	64746	ACBD3	1	0	1.00E+00	
90	4041	LRP5	1	0	1.00E+00	
91	9129	PRPF3	1	0	1.00E+00	
92	22926	ATF6	1	0	1.00E+00	-
93	9482	STX8	4	0	1.00E+00	
94	55755	CDK5RAP2	2	0	1.00E+00	
95	4591	TRIM37	1	0	1.00E+00	
96	22827	PUF60	4	0	1.00E+00	
97	3840	KPNA4	1	0	1.00E+00	
98	4163	MCC	1	0	1.00E+00	
99	335	APOA1	5	0	1.00E+00	
100	4780	NFE2L2	2	0	1.00E+00	
101	2804	GOLGB1	2	0	1.00E+00	

+	positive effect on MHV-68 replication
-	negative effects on MHV-68 replication
Toxic	Toxic effects from siRNA treatment
