# Supplementary material for: An Integrated Approach to Elucidate the Intra-Viral and Viral-Cellular Protein Interaction Networks of a Gamma-Herpesvirus
Source: PLoS Pathog. 2011 Oct 20;7(10):e1002297. doi: 10.1371/journal.ppat.1002297 (PMC3197595; doi:10.1371/journal.ppat.1002297)
Supplement: Table S4 — Priority rank of cellular proteins based on the merged EBV- [24] and MHV-68-PPI hit list. (PDF) [file ppat.1002297.s012.pdf]

Table S4. Priority rank of cellular proteins based on the merged EBV (23) and MHV-68 PPI hit list

GeneID	Name	score(merged)	p-val(merged)	Score	p-val	Virus
7531	YWHAE	120	1.02E-04	81	2.19E-05	MHV-68
7329	UBE2I	115	2.94E-04	75	1.87E-04	MHV-68
1387	CREBBP	115	2.94E-04	72	4.84E-04	MHV-68
7329	UBE2I	115	2.94E-04	41	4.11E-03	EBV
1499	CTNNB1	112	5.19E-04	70	8.55E-04	MHV-68
8554	PIAS1	103	2.40E-03	65	2.88E-03	MHV-68
3309	HSPA5	102	2.78E-03	59	8.40E-03	MHV-68
5499	PPP1CA	97	5.24E-03	30	3.64E-02	EBV
2316	FLNA	93	8.18E-03	52	2.05E-02	MHV-68
7251	TSG101	90	1.09E-02	24	8.08E-02	EBV
4790	NFKB1	89	1.20E-02	29	4.19E-02	EBV
10527	IPO7	84	1.79E-02	54	1.62E-02	MHV-68
8936	WASF1	76	3.09E-02	50	2.55E-02	MHV-68
80342	TRAF3IP3	74	3.49E-02	27	5.55E-02	EBV
8723	SNX4	73	3.70E-02	26	6.31E-02	EBV
163	AP2B1	72	3.93E-02	24	8.08E-02	EBV
81	ACTN4	70	4.42E-02	32	1.19E-01	MHV-68
81	ACTN4	70	4.42E-02	18	1.56E-01	EBV
51517	NCKIPSD	70	4.42E-02	27	5.55E-02	EBV
3838	KPNA2	66	5.54E-02	18	1.56E-01	EBV
8678	BECN1	64	6.18E-02	41	5.90E-02	MHV-68
26512	INTS6	63	6.52E-02	40	6.41E-02	MHV-68
54764	ZRANB1	63	6.52E-02	19	2.72E-01	MHV-68
3276	HRMT1L2	63	6.52E-02	13	2.53E-01	EBV
5684	PSMA3	58	8.41E-02	18	1.56E-01	EBV
6125	RPL5	57	8.83E-02	21	2.41E-01	MHV-68
5315	PKM2	56	9.27E-02	4	5.68E-01	EBV
2335	FN1	55	9.73E-02	24	2.01E-01	MHV-68
2335	FN1	55	9.73E-02	15	2.10E-01	EBV
6720	SREBF1	54	1.02E-01	19	2.72E-01	MHV-68
4000	LMNA	53	1.07E-01	17	3.05E-01	MHV-68
142	PARP1	52	1.12E-01	60	7.17E-03	MHV-68
2202	EFEMP1	51	1.17E-01	6	4.72E-01	EBV
29760	BLNK	51	1.17E-01	5	5.17E-01	EBV
8470	SORBS2	50	1.22E-01	11	4.31E-01	MHV-68
6386	SDCBP	48	1.34E-01	15	3.42E-01	MHV-68
8661	EIF3A	45	1.52E-01	24	2.01E-01	MHV-68
9276	COPB2	45	1.52E-01	16	3.23E-01	MHV-68
708	C1QBP	45	1.52E-01	5	5.17E-01	EBV
4926	NUMA1	43	1.66E-01	25	1.89E-01	MHV-68
6717	SRI	43	1.66E-01	14	2.30E-01	EBV
3690	ITGB3	42	1.72E-01	26	1.77E-01	MHV-68
335	APOA1	41	1.80E-01	0	1.00E+00	MHV-68
22919	MAPRE1	41	1.80E-01	7	4.32E-01	EBV
5525	PPP2R5A	40	1.87E-01	44	4.53E-02	MHV-68
2597	GAPDH	39	1.94E-01	25	7.16E-02	EBV
7846	TUBA3	39	1.94E-01	24	8.08E-02	EBV
10987	COPS5	37	2.11E-01	43	4.95E-02	MHV-68
5094	PCBP2	37	2.11E-01	22	2.28E-01	MHV-68
10919	EHMT2	37	2.11E-01	2	8.01E-01	MHV-68
2801	GOLGA2	34	2.36E-01	4	6.66E-01	MHV-68
6672	SP100	34	2.36E-01	25	7.16E-02	EBV

1639	DCTN1	33	2.45E-01	41	5.90E-02	MHV-68
10318	TNIP1	33	2.45E-01	25	1.89E-01	MHV-68
3308	HSPA4	32	2.55E-01	36	8.83E-02	MHV-68
9231	DLG5	32	2.55E-01	18	2.88E-01	MHV-68
5713	PSMD7	32	2.55E-01	15	3.42E-01	MHV-68
79027	ZNF655	32	2.55E-01	4	6.66E-01	MHV-68
87	ACTN1	31	2.65E-01	39	6.95E-02	MHV-68
6711	SPTBN1	31	2.65E-01	36	8.83E-02	MHV-68
51720	RAP80	31	2.65E-01	34	1.03E-01	MHV-68
57530	CGN	31	2.65E-01	20	2.56E-01	MHV-68
2243	FGA	31	2.65E-01	1	8.53E-01	MHV-68
87	ACTN1	31	2.65E-01	22	1.02E-01	EBV
1195	CLK1	31	2.65E-01	7	4.32E-01	EBV
5978	REST	30	2.75E-01	31	1.27E-01	MHV-68
8976	WASL	29	2.86E-01	34	1.03E-01	MHV-68
9994	CASP8AP2	29	2.86E-01	33	1.10E-01	MHV-68
7431	VIM	29	2.86E-01	19	1.41E-01	EBV
22827	PUF60	28	2.96E-01	0	1.00E+00	MHV-68
3856	KRT8	26	3.19E-01	33	1.10E-01	MHV-68
5093	PCBP1	26	3.19E-01	30	1.36E-01	MHV-68
6383	SDC2	26	3.19E-01	14	3.63E-01	MHV-68
3320	HSP90AA1	25	3.31E-01	25	1.89E-01	MHV-68
23471	TRAM1	25	3.31E-01	12	4.07E-01	MHV-68
7186	TRAF2	25	3.31E-01	13	2.53E-01	EBV
3183	HNRNPC	24	3.44E-01	28	1.55E-01	MHV-68
10142	AKAP9	23	3.57E-01	37	8.16E-02	MHV-68
23384	KIAA0376	23	3.57E-01	14	3.63E-01	MHV-68
3172	HNF4A	22	3.70E-01	12	4.07E-01	MHV-68
7185	TRAF1	22	3.70E-01	16	1.90E-01	EBV
2065	ERBB3	20	3.98E-01	22	2.28E-01	MHV-68
84433	CARD11	19	4.13E-01	25	1.89E-01	MHV-68
7184	HSP90B1	19	4.13E-01	17	3.05E-01	MHV-68
4055	LTBR	19	4.13E-01	3	7.15E-01	MHV-68
9541	CIR	18	4.28E-01	6	4.72E-01	EBV
6122	RPL3	17	4.44E-01	12	2.77E-01	EBV
10771	ZMYND11	17	4.44E-01	1	7.91E-01	EBV
23126	POGZ	16	4.61E-01	18	2.88E-01	MHV-68
4001	LMNB1	16	4.61E-01	13	2.53E-01	EBV
6709	SPTAN1	14	4.96E-01	15	3.42E-01	MHV-68
667	DST	14	4.96E-01	9	4.84E-01	MHV-68
6830	SUPT6H	13	5.15E-01	14	3.63E-01	MHV-68
8655	DNCL1	13	5.15E-01	12	2.77E-01	EBV
10989	IMMT	13	5.15E-01	8	3.96E-01	EBV
11117	EMILIN1	12	5.35E-01	19	2.72E-01	MHV-68
10605	PAIP1	11	5.55E-01	17	3.05E-01	MHV-68
8887	TAX1BP1	11	5.55E-01	17	3.05E-01	MHV-68
7917	BAT3	11	5.55E-01	9	4.84E-01	MHV-68
7917	BAT3	11	5.55E-01	6	4.72E-01	EBV
7791	ZYX	11	5.55E-01	2	7.12E-01	EBV
4678	NASP	10	5.78E-01	12	4.07E-01	MHV-68
6117	RPA1	10	5.78E-01	10	4.57E-01	MHV-68
65125	WNK1	10	5.78E-01	2	8.01E-01	MHV-68
4925	NUCB2	10	5.78E-01	7	4.32E-01	EBV
3912	LAMB1	10	5.78E-01	6	4.72E-01	EBV
9971	NR1H4	9	6.01E-01	7	5.45E-01	MHV-68

4628	MYH10	8	6.28E-01	6	5.80E-01	MHV-68
1998	ELF2	8	6.28E-01	4	5.68E-01	EBV
7073	TIAL1	7	6.56E-01	7	5.45E-01	MHV-68
5927	KDM5A	7	6.56E-01	7	5.45E-01	MHV-68
7316	UBC	7	6.56E-01	7	5.45E-01	MHV-68
3164	NR4A1	7	6.56E-01	3	6.27E-01	EBV
11335	CBX3	7	6.56E-01	2	7.12E-01	EBV
9318	COPS2	6	6.90E-01	6	5.80E-01	MHV-68
5693	PSMB5	6	6.90E-01	3	7.15E-01	MHV-68
3043	HBB	6	6.90E-01	4	5.68E-01	EBV
23062	GGA2	5	7.23E-01	12	4.07E-01	MHV-68
7402	UTRN	5	7.23E-01	9	4.84E-01	MHV-68
10788	IQGAP2	5	7.23E-01	6	5.80E-01	MHV-68
10788	IQGAP2	5	7.23E-01	4	5.68E-01	EBV
84726	BAT2L	4	7.76E-01	5	6.18E-01	MHV-68
51100	SH3GLB1	4	7.76E-01	2	7.12E-01	EBV
9454	HOMER3	4	7.76E-01	2	7.12E-01	EBV
9463	PRKCABP	4	7.76E-01	0	1.00E+00	EBV
3843	IPO5	3	8.13E-01	5	6.18E-01	MHV-68
9416	DDX23	3	8.13E-01	3	7.15E-01	MHV-68
25801	GCA	3	8.13E-01	2	7.12E-01	EBV
23365	ARHGEF12	2	8.95E-01	6	5.80E-01	MHV-68
213	ALB	2	8.95E-01	3	7.15E-01	MHV-68
4502	MT2A	2	8.95E-01	1	8.53E-01	MHV-68
5359	PLSCR1	2	8.95E-01	2	7.12E-01	EBV
29950	SERTAD1	2	8.95E-01	0	1.00E+00	EBV
4188	MDFI	2	8.95E-01	0	1.00E+00	EBV
7450	VWF	2	8.95E-01	0	1.00E+00	EBV
6249	RSN	1	9.22E-01	2	8.01E-01	MHV-68
25831	HECTD1	1	9.22E-01	1	8.53E-01	MHV-68
4924	NUCB1	1	9.22E-01	1	7.91E-01	EBV
10616	C20orf18	1	9.22E-01	0	1.00E+00	EBV
10197	PSME3	1	9.22E-01	0	1.00E+00	EBV
2244	FGB	0	1.00E+00	2	8.01E-01	MHV-68
10241	CALCOCO2	0	1.00E+00	1	8.53E-01	MHV-68
23499	MACF1	0	1.00E+00	1	8.53E-01	MHV-68
64746	ACBD3	0	1.00E+00	0	1.00E+00	MHV-68
4041	LRP5	0	1.00E+00	0	1.00E+00	MHV-68
9129	PRPF3	0	1.00E+00	0	1.00E+00	MHV-68
22926	ATF6	0	1.00E+00	0	1.00E+00	MHV-68
9482	STX8	0	1.00E+00	0	1.00E+00	MHV-68
55755	CDK5RAP2	0	1.00E+00	0	1.00E+00	MHV-68
4591	TRIM37	0	1.00E+00	0	1.00E+00	MHV-68
3840	KPNA4	0	1.00E+00	0	1.00E+00	MHV-68
4163	MCC	0	1.00E+00	0	1.00E+00	MHV-68
4780	NFE2L2	0	1.00E+00	0	1.00E+00	MHV-68
2804	GOLGB1	0	1.00E+00	0	1.00E+00	MHV-68
55072	RNF31	0	1.00E+00	1	7.91E-01	EBV
30008	EFEMP2	0	1.00E+00	0	1.00E+00	EBV
59277	NTN4	0	1.00E+00	0	1.00E+00	EBV
11030	RBPMS	0	1.00E+00	0	1.00E+00	EBV
9353	SLIT2	0	1.00E+00	0	1.00E+00	EBV
2896	GRN	0	1.00E+00	0	1.00E+00	EBV
1075	CTSC	0	1.00E+00	0	1.00E+00	EBV
5598	MAPK7	0	1.00E+00	0	1.00E+00	EBV

10516	FBLN5	0	1.00E+00	0	1.00E+00	EBV
972	CD74	0	1.00E+00	0	1.00E+00	EBV
81628	TSC22D4	0	1.00E+00	0	1.00E+00	EBV
6124	RPL4	0	1.00E+00	0	1.00E+00	EBV
