# Supplementary material for: An Integrated Approach to Elucidate the Intra-Viral and Viral-Cellular Protein Interaction Networks of a Gamma-Herpesvirus
Source: PLoS Pathog. 2011 Oct 20;7(10):e1002297. doi: 10.1371/journal.ppat.1002297 (PMC3197595; doi:10.1371/journal.ppat.1002297)
Supplement: Table S5 — Yeast strains used in this study. (PDF) [file ppat.1002297.s013.pdf]

Table S5. Yeast strains used in this study.

Yeast strain	Genotype	Reference
PJ69-4a	*MAT*a *trp1-901 leu2-3,112 ura3-52 his3-200 gal4Δ gal80Δ LYS2::GAL1-HIS3 GAL2-ADE2 met2::GAL7-lacZ*	(Calderwood et al. 2007)
PJ69-4α	*MATα trp1-901 leu2-3,112 ura3-52 his3-200 gal4Δ gal80Δ LYS2::GAL1-HIS3 GAL2-ADE2 met2::GAL7-lacZ*	(James et al. 1996)
R2HMet	*MATa ura3-52 ade2-101 trp1-901 leu2-3,112 his3-200 met2Δ::hisG gal4Δ gal80Δ*	(LaCount et al. 2005)
BK100	*MATa ura3-52 ade2-101 trp1-901 leu2-3,112 his3-200 gal4Δ gal80Δ GAL2-ADE2 LYS2::GAL1-HIS3 met2::GAL7-lacZ*	[24]
